# Parameter estimation and uncertainty quantification using information geometry

**DOI:** 10.1098/rsif.2021.0940

**Published:** 2022-04-27

**Authors:** Jesse A. Sharp, Alexander P. Browning, Kevin Burrage, Matthew J. Simpson

**Affiliations:** ^1^ School of Mathematical Sciences, Queensland University of Technology, Brisbane, Queensland, Australia; ^2^ ARC Centre of Excellence for Mathematical and Statistical Frontiers, Queensland University of Technology, Brisbane, Queensland, Australia; ^3^ Department of Computer Science, University of Oxford, Oxford, UK; ^4^ Centre for Data Science, Queensland University of Technology, Brisbane, Queensland, Australia

**Keywords:** inference, likelihood, population models, logistic growth, epidemic models

## Abstract

In this work, we: (i) review likelihood-based inference for parameter estimation and the construction of confidence regions; and (ii) explore the use of techniques from information geometry, including geodesic curves and Riemann scalar curvature, to supplement typical techniques for uncertainty quantification, such as Bayesian methods, profile likelihood, asymptotic analysis and bootstrapping. These techniques from information geometry provide data-independent insights into uncertainty and identifiability, and can be used to inform data collection decisions. All code used in this work to implement the inference and information geometry techniques is available on GitHub.

## 1. Introduction

Computational and mathematical models are versatile tools, providing valuable insight into complex processes in the life sciences. Models can further our understanding of mechanisms and processes, facilitate development and testing of hypotheses, guide experimentation and data collection and aid design of targeted interventions [1–5]. However, there are considerable challenges associated with calibrating these models to data. For example, models need to be sufficiently sophisticated to adequately reflect the behaviour of the underlying system, while ideally admitting *identifiable* parameters that are interpretable and that can be estimated from available or obtainable data [6,7]. Further, available data can be limited and often are not collected for the express purpose of parameter estimation; data may be noisy or incomplete, or may not provide the level of detail or sample size required to obtain precise parameter estimates [8–12].

Owing to the challenges associated with parameter estimation, we are often interested in not only point estimates, but also the associated uncertainty [13–15]. Quantifying and interpreting this uncertainty establishes a level of confidence in parameter estimates and, by extension, in the insights derived from the model. Further, this uncertainty quantification can give insights into *identifiability*: whether the information in a dataset can be used to infer unique or sufficiently precise parameter estimates for a given model [16]. Often we are concerned with both *structural identifiability* and *practical identifiability* [17–21]. Structural identifiability can be thought of as a property of the underlying model structure and parametrization, and refers to whether it is theoretically possible to determine unique parameter values, given an infinite amount of perfect noise-free data [16,22,23]. Structural identifiability requires that unique parameter combinations precipitate distinct model outputs. Structural identifiability occurs if and only if the Fisher information matrix, which we soon discuss, is of full rank [24]. By contrast, practical identifiability is less well defined, and depends on the quality and quantity of data available and existing knowledge of the parameters [22]. In the context of profile-likelihood methods, practical non-identifiability can manifest as contours of the log-likelihood function that do not admit closed levels; the log-likelihood does not reach a predetermined statistical threshold within the physical parameter regime [25]. If a model is not structurally identifiable, it cannot be practically identifiable.

Practical non-identifiability may be addressed through improving either data quantity or data quality [19,22]. Data quantity can be improved by increasing the number of observations, such as by making additional observations at different time points. Data quality may be improved through reducing the noise present in the data; for example, by obtaining a dataset with reduced measurement error or repeating measurements across experiments [26,27]. It is also possible to resolve practical non-identifiability through incorporating existing knowledge about parameters, such as physical constraints or information established in previous studies; or specifically in the Bayesian inferential framework, through informative priors [28]. Addressing structural non-identifiability is more challenging; for example, this may necessitate a change to the underlying model structure [20,27,29]. Uncertainty quantification takes many forms, with common examples, including Bayesian methods, profile likelihood, asymptotic analysis and bootstrapping [8,12,30–32]. Bayesian methods are widely used for parameter estimation and uncertainty quantification, with Bayesian computation being employed throughout the mathematical biology and systems biology literature. Broadly, these methods involve repeated sampling of parameter values from a prior distribution and invoking Bayes' theorem to approximate the *posterior* distribution; the posterior distribution describes knowledge about the probability of parameter combinations after taking into account the observed data and any prior information [22,32]. Well-known approaches include rejection sampling, Markov chain Monte Carlo (MCMC) and sequential Monte Carlo (SMC) or particle filtering. In rejection sampling, parameters drawn from a prior distribution are used to simulate the model. Simulated data are compared with the observed data based on some distance metric; if this metric is within a prescribed tolerance, the parameters are accepted as a sample from the approximate posterior distribution, otherwise they are rejected [30,33]. Rejection sampling can be computationally expensive as the rejection rate can be significant with an uninformative prior [34,35]. In MCMC, the parameter space is sampled following a Markov chain—a memoryless stochastic process where the probability of the next state depends only on the previous state [36]—with a stationary distribution corresponding to the posterior distribution. Samples are accepted or rejected based on the relative likelihood between the current configuration and proposed sample [11,32,37,38]. For SMC, rejection sampling can be used to produce an initial coarse approximation of the posterior distribution. This coarse approximation informs further (sequential) sampling efforts in the region of parameter space corresponding to high likelihood, reducing the rejection rate when compared with rejection sampling alone [11,34,39]. MCMC and SMC approaches can offer significantly improved efficiency in comparison with rejection sampling [32,34], but both involve specifying hyperparameters and these choices are not always obvious. In situations where the likelihood function is intractable or not easily evaluated, approximate Bayesian computation (ABC) provides a range of related likelihood-free methods for estimating posterior distributions [40]. Popular approaches include ABC rejection sampling [35,39–42], ABC MCMC [43–45] and ABC SMC [11,34]; we do not focus on ABC methods here, as the approaches we explore in this work are applied to problems with tractable likelihoods. We direct interested readers to the wealth of information in the references provided.

For Bayesian inference methods, uncertainty can be quantified based on features such as the coefficient of variation and probability intervals of the posterior distribution [12]. There are a variety of approaches for uncertainty quantification for frequentist inference methods. In profile likelihood, a parameter of interest is varied over a fixed set of values, while re-estimating the other parameters; this provides insight into identifiability and uncertainty [1]. In asymptotic analysis, confidence regions can be constructed based on local information via a Taylor expansion of the Fisher information about the maximum likelihood estimate (MLE) [8,25]. In bootstrapping, data are repeatedly sampled and parameter estimates are computed from the samples; these estimates are used to construct confidence intervals [31].

Through the geometric approaches we review in this work, more akin to traditional approaches for sensitivity analysis [14,46,47], we explore the curvature of the parameter space through an information metric induced by the likelihood function. Whereas likelihood-based approximate confidence regions provide insight into specific level curves of the likelihood function—the levels of which depend on an asymptotic large sample argument [36]—this geometric approach provides insight into the *shape* and sensitivity of the parameter space. For example, we compute *geodesic curves* that describe the geometric relationship between distributions with different parameters [48]; and explore the *scalar curvature* throughout parameter spaces. We review ideas from *information geometry* in the context of inference and uncertainty quantification; not with a view to replacing established methods such as profile likelihood, asymptotic analysis, bootstrapping and Bayesian methods [8,12,31,32], but rather to supplement them where additional insight may prove useful.

Information geometry is a branch of mathematics connecting aspects of information theory including probability theory and statistics with concepts and techniques in differential geometry [49]. In this exposition, we seek to outline only the key concepts required to understand the information geometric analysis in this work. However, we note that more thorough and rigorous treatments of the concepts introduced in this section, and mathematical foundations of information geometry, can be found in texts and surveys such as [49–51]. Central to the information geometry ideas explored in this work is the concept of a *statistical manifold*; an abstract geometric representation of a distribution space, or a Riemannian manifold consisting of points that correspond to probability distributions, with properties that we later discuss. For example, the set of normal distributions parametrized by mean, *μ*, and standard deviation, *σ* > 0,

1.1 $$p(x;\mu ,\sigma )=\frac{1}{\sigma \sqrt{2\pi }}\exp\left[-\frac{{\left(x-\mu \right)}^{2}}{2\sigma^{2}}\right],\;x\in R,$$

can be thought of as a two-dimensional surface with coordinates (*μ*, *σ*) [50]. In this work, we will use ***θ*** to refer to the parameters of interest that we seek to estimate; i.e. ***θ*** = (*μ*, *σ*) for the univariate normal distribution with unknown mean and standard deviation. In §3, we consider various combinations of components of ***θ***, including model parameters, variability in observations characterized by a separate observation noise model, and initial conditions associated with a differential equation-based process model. When referring to all possible parameters, rather than solely the unknown parameters to be estimated, we denote this Θ.

In applications where we consider multiple datasets, or different candidate models or candidate parameter values, we are interested in methods of comparing distributions. A well-known measure for comparing a probability distribution, *P*, with another, *Q*, is the Kullback–Leibler (KL) divergence from *P* to *Q*, denoted $D_{\mathrm{KL}}(P,Q)$ [52]. The KL divergence, or *relative entropy*, can be computed as [52]

1.2 $$D_{\mathrm{KL}}(P,Q)=\int p(x)\log\frac{\;p(x)}{q(x)}\;dx=E_{p}\left[\log\frac{\;p(x)}{q(x)}\right],$$

where *p*(*x*) and *q*(*x*) are the respective probability density functions of *P* and *Q*. Consider two sets of parameters, θ* and $\;\hat{\theta }$; let log(*p*(*x*)) = log(*p*(*x*|θ*)) = ℓ(θ*) and $\log(q(x))=\log(p(x|\;\hat{\theta }))=\ell (\;\hat{\theta })$, where ℓ( · ) denotes the log-likelihood, discussed in detail in §2. If *p*(*x*|θ*) is the true distribution and $p(x|\;\hat{\theta })$ is our estimate, then (1.2) is the expected log-likelihood ratio and the relationship between MLE and KL divergence becomes evident; maximizing the likelihood is equivalent to minimizing the KL divergence [53].

An issue with the KL divergence is asymmetry; $D_{\mathrm{KL}}(P,Q)\neq D_{\mathrm{KL}}(Q,P)$. It is not necessarily obvious in a given situation which orientation of the KL divergence will most appropriately inform decisions such as model selection [54]. Owing to the aforementioned asymmetry, and its failure to satisfy the triangle inequality, the KL divergence is not a *metric*—it is not a measure of distance in a differential geometric sense—on a given manifold [50]. One means of addressing this asymmetry is through devising various symmetrized forms of the KL divergence to inform model selection criteria [54]. Alternatively, we may approach the issue from a geometric perspective. It is natural to think of geometry in terms of objects or shapes in Euclidean, or *flat*, space. Euclidean space is characterized by orthonormal basis vectors; the standard basis in three dimensions being ***e***_1_ = (1, 0, 0)^T^, ***e***_2_ = (0, 1, 0)^T^, ***e***_3_ = (0, 0, 1)^T^, where superscript T denotes the transpose. In the *n*-dimensional orthonormal basis, we can compute the squared infinitesimal distance between the points S and S+ds, where d*s* has components d*s*_*i*_, as [55]

1.3 $$\|ds{\|}^{2}=\sum_{i=1}^{n}{\left(ds_{i}\right)}^{2}.$$

Differential geometry extends ideas from Euclidean geometry to manifolds. Manifolds are topological spaces that resemble flat space about each individual point in the space; they can be considered *locally flat*, but have a different topology globally. The sphere is a classic example, whereby points on the surface are locally topologically equivalent to two-dimensional Euclidean space, but globally the sphere is curved and has a compact topology; it is bounded and closed [56]. In particular, we are interested in Riemannian manifolds; differentiable manifolds—sufficiently locally smooth that our typical notions of calculus remain valid—upon which we are able to measure geometric quantities such as distance, through the existence of a Riemannian metric on the tangent space of the manifold, that generalizes the notion of an inner product from Euclidean geometry [57].

A Riemannian metric is a smooth covariant 2-tensor field; on a differentiable manifold *M*, the Riemannian metric is given by an inner product on the tangent space of the manifold, *T*_*p*_*M*, which depends smoothly on the base point *p* [57,58]. A tangent space can be thought of as a multidimensional generalization of a tangent plane to a three-dimensional surface. Each point *p* on a manifold is associated with a distinct tangent space. An *n*-dimensional manifold has infinitely many *n*-dimensional tangent spaces; the collection of these tangent spaces is referred to as the tangent bundle of the manifold. On a manifold each tangent space can have different basis vectors, in contrast to Euclidean geometry, where tangent vectors at any point have the same basis vectors. A consequence of the distinct basis vectors of tangent spaces on manifolds is that tangent vectors at different points on the manifold cannot be directly added or subtracted. Introducing an *affine connection* on the manifold connects nearby tangent spaces, such that the manifold looks infinitesimally like Euclidean space, which facilitates differentiation of tangent vectors [59]. Formally, we introduce the unique, torsion-free Levi–Civita connection, ∇; this is an affine connection on the Riemannian manifold that yields isometric parallel transport, such that inner products between tangent vectors, defined by the metric, are preserved [60]. The coefficients of this connection are the Christoffel symbols, which we discuss further in §2. Readers are directed to [59–61] for further detail regarding the Levi–Civita connection and how it relates to other concepts discussed in this work. A manifold equipped with such a connection and a Riemann metric is a Riemann manifold.

Metric tensors can be thought of as functions that facilitate computation of quantities of interest such as distances on a manifold. A metric matrix with elements *g*_*ij*_, *G* = [*g*_*ij*_], is positive definite and symmetric [57]. The metric matrix defines an inner product between *u* and *v* as 〈*u*, *v*〉_*G*_ = *u*^T^*Gv*, and the length of *u* as $\|u{\|}_{G}=\sqrt{{\left\langle u,u\right\rangle }_{G}}$ [62]. On a Riemannian manifold, we consider a generalization of the square of the infinitesimal distance element (1.3), appropriate for non-orthonormal bases [55], given by

$$\|ds{\|}^{2}=\sum_{i,j=1}^{n}g_{ij}\;ds_{i}\;ds_{j}.$$

Foundational to information geometry is the notion that the Fisher information matrix defines a Riemannian metric on a statistical manifold [63]. The Fisher information, denoted I(θ), describes the expected curvature of the log-likelihood and gives information about the precision and variance of parameter estimates. Therefore, I(θ) can incorporate information about both the curvature induced by the data through the observation process and the curvature induced by parameter sensitivities through a mathematical model that links parameter estimates to data. In the examples we consider, deterministic model predictions are connected to the data through the probabilistic observation process, yielding a general formula for the Fisher information [64]

1.4 $$I(\theta )=NJ{\left(\theta \right)}^{T}\overset{\begin{array}{rl} & \mathrm{Curvature}\\ & \;\mathrm{induced}\\ & \;\mathrm{by}\;\mathrm{data} \end{array}}{\overbrace{O(m)}}\underset{\begin{array}{rl} & \mathrm{Curvature}\;\mathrm{induced}\\ & \;\mathrm{by}\;\mathrm{parameter}\\ & \;\;\mathrm{sensitivities} \end{array}}{\underbrace{J(\theta )}}.$$

Here, we denote O(m) the Fisher information matrix of the observation process, given a model, **m** = **m**(***θ***), where **J**(***θ***) is the Jacobian of the model with respect to the parameters. The number of independent, identically distributed (iid) observations in the likelihood is given by *N*; with statistical independence, the Fisher information is additive [65].

Expression (1.4) highlights a link between sensitivity analysis, structural identifiability and practical identifiability [66]. For sensitivity analysis and structural identifiability, only the curvature of the model space is studied through **J**(***θ***). In practical identifiability analysis, the sensitivity of the model is linked to the data through an observation process, and the curvature of the parameter space is studied through, for example, I(θ).

In this review, we present and explore fundamental techniques in inference and information geometry, including confidence regions, geodesic curves and scalar curvature. Through application to standard distributions and canonical models in the life sciences, including population growth processes and epidemic transmission, we demonstrate how these techniques can be combined to provide additional insights into parameter estimation and uncertainty quantification. Starting with parameter estimates inferred from real data, we use mathematical models to generate synthetic data with different numbers of observations and at varying points in time, to explore the impact that these aspects have on the inference and information geometry results. Specifically, we consider univariate and multivariate normally distributed observation processes; linear, exponential and logistic models of population growth; and the classical susceptible, infectious, recovered (SIR) model of epidemic transmission [67,68]. Although the examples considered in this work are based on ordinary differential equation (ODE) process models drawn from the life sciences, the techniques we consider are general and can be applied in the context of parameter estimation and uncertainty quantification in any discipline and for other model formulations.

By considering standard distributions and canonical models, we are able to explore the inference and information geometry techniques through a series of examples with incremental increases in complexity. Through this approach, we consider the techniques as applied to both linear and nonlinear ODE models, coupled nonlinear ODE systems and data with both one and many observed variables. We consider cases where model parameters, initial conditions and the standard deviation of the data are to be estimated from data. The inference and information geometry techniques considered in this work are general, and can be applied far more widely than the examples we consider here. To improve the accessibility of these methods, code used to implement the inference and information geometry techniques applied in this work is written in the open source Julia language [69] and is available on GitHub.

In §2, we describe the inference and information geometry methods implemented in this work, including maximum likelihood estimation, profile-likelihood-based approaches, geodesic curves and scalar curvature calculations. Results of applying these techniques to univariate and multivariate normal distributions, linear, exponential and logistic growth models and the SIR model are presented in §3. We discuss the utility of these techniques and identify opportunities for extension and further consideration in §4.

## 2. Methods

Here we describe the parameter inference and information geometry methods used to produce results in this work. We also describe the numerical methods used to implement these techniques. The techniques we discuss in this section readily generalize to parameter spaces with an arbitrary number of dimensions, so we discuss the techniques here for arbitrary dimensions. However, for the sake of exploring the techniques through visualization in §3, we restrict ourselves to two-dimensional manifolds. In context, this means we consider only two parameters to be inferred in any given example, treating other parameters as known and fixed; for example, as if they are drawn from prior knowledge or pre-estimated.

Although we consider deterministic mathematical models, data used to estimate parameters can exhibit significant variability. We follow a standard approach and assume that the mathematical models describe the expected behaviour, and that our observations are normally distributed about this expected behaviour [18]. This allows us to think about a statistical model, ***m***(***θ***, *t*), in terms of its expected behaviour, ***μ****,* and the standard deviation of the observations, *σ*,

m(θ,t)=(μ(θ,t),σ(θ,t)).

We restrict the examples in this work to cases where *σ* is constant, setting *σ*(***θ***, *t*) = *σ*. In this work we focus on the most commonly employed additive noise model [5,11,18,19,27]. Additive noise implies that the variance of the data is independent of the mean behaviour. In cases where variance scales with mean behaviour, multiplicative noise may be more appropriate. The information geometric methods presented here are applicable in cases where the Fisher information can be obtained, including models with multiplicative noise and parameter- or time-dependent standard deviation. However, obtaining the Fisher information is a separate challenge, and can be difficult when considering different process and noise models.

### 2.1. Parameter inference

In this work, parameter estimates are inferred from data following a standard maximum log-likelihood-based approach. We make observations at *L* time points, *T* = (*t*_1_, *t*_2_, …, *t*_*L*_). At each time point, we make *N* observations, $X=(x_{1}(T),x_{2}(T),\ldots ,x_{N}(T))$. With this notation, the log-likelihood function is

2.1 $$\ell (\theta ;X)=\sum_{\;j=1}^{L}\sum_{i=1}^{N}\log f(x_{i}(t_{j});\mu (\theta ,t_{j}),\sigma^{2}),$$

where *f*(**x**; ***μ***, *σ*^2^) is the probability density function associated with our observation process. In this work, we hold *N* constant across time points, though non-constant *N* is easily incorporated into equation (2.1) as *N*_*j*_. The likelihood function can be thought of as the joint probability density of all the data for a given set of parameters. In examples where *σ* is unknown, we treat *σ* as an element of ***θ***, but note that the expected model behaviour is independent of *σ*. The MLE is the point estimate, $\;\hat{\theta }$, that satisfies

2.2 $$\hat{\theta }=\underset{\theta }{\arg\;\max}\ell (\theta ;X),$$

where arg max( · ) returns the argument, ***θ***, that maximizes ℓ(θ;X) in (2.2). The associated maximum log-likelihood is $\ell (\hat{\theta })$. MLEs of the parameters of interest are obtained by solving (2.2) numerically as outlined later in §2. For an iid sample from a univariate normal distribution, $N(\mu ,\sigma^{2})$, maximizing the likelihood function of *μ* is equivalent to performing least-squares estimation [22], although having access to the likelihood function facilitates uncertainty quantification.

Presenting confidence regions alongside MLEs enhances our interpretation of the likelihood function, while still acknowledging that the estimates carry uncertainty [36]. We apply a probability-based log-likelihood approach when constructing confidence regions for model parameters. From Wilks’ theorem [36], asymptotically as *N* → ∞, an approximate *α*-level confidence region is given by

2.3 $$\left\{\theta \;:\;\ell (\theta )\geq \ell (\;\hat{\theta })-\frac{\Delta_{\nu ,\alpha }}{2}\right\},$$

where Δ_*ν*,*α*_ is the *α*th-quantile of the *χ*^2^(*ν*) distribution, with *ν* degrees of freedom [1]. In this work, the degrees of freedom correspond to the number of parameters of interest, i.e. *ν* = dim(***θ***). To enable comparison between different datasets and models in §3, we consider the normalized log-likelihood, $\hat{\ell }(\theta )=\ell (\theta )-\ell (\hat{\theta })$. This forms the basis for log-likelihood ratio-based hypothesis tests [36]. The normalized log-likelihood is zero at the MLE: $\hat{\ell }(\hat{\theta })\equiv 0$.

### 2.2. Information geometry

As outlined in §1, the Fisher information describes the curvature of the log-likelihood function. It describes how much information a random variable, *X*, contains about a parameter, ***θ***. For unbiased estimators, the inverse of the Fisher information provides a lower bound on the covariance matrix, via the Cramér–Rao inequality [70]. Formally, the Fisher information is the covariance of the score, where the score is defined as the partial derivative of the log-likelihood with respect to ***θ*** [36,64]. The Fisher information matrix can be written as [36,71]

2.4 $${\left[I(\theta )\right]}_{ij}=E_{X}\left[\left(\frac{\partial }{\partial \theta_{i}}\log f\;(X;\theta )\right)\left(\frac{\partial }{\partial \theta_{j}}\log f\;(X;\theta )\right)\right].$$

We can recover our expression for the Fisher information in equation (1.4) from equation (2.4), by considering how equation (2.4) changes under reparametrization and via application of the chain-rule for differentiation [64]. With observations at *L* unique times, *T* = (*t*_1_, *t*_2_, …, *t*_*L*_), we can think of a model as a mapping between the parameters and the outputs that we can observe,

2.5 $$m(\theta )\;:\;\theta \to ((\mu_{1}(\theta ,t_{1}),\sigma ),(\mu_{2}(\theta ,t_{2}),\sigma ),\ldots ,(\mu_{L}(\theta ,t_{L}),\sigma )).$$

We consider some examples where *σ* is unknown and is estimated as a part of the analysis; in these instances *σ* ∈ ***θ***, however we express *σ* explicitly in the mapping presented in (2.5) to emphasize that it behaves somewhat differently from a model parameter. The expected behaviour of the model does not depend on *σ*, and variability in the data maps directly to *σ*. In all the examples we consider, *σ* is constant. This could be extended to incorporate variability dependent on the expected behaviour; for example, logistic growth with standard deviation that depends on the population density [72]. In the mapping, this could be expressed as *σ*(***μ***(***θ***, *t*)).

Following equation (1.4), we can form the Fisher information as a combination of the Fisher information matrix of the observation process, O(m), and the Jacobian of the model with respect to the parameters, **J**(***θ***). From (2.5), with *ν* unknown parameters (dim⁡(θ)=ν), we can view the model Jacobian as

2.6 $$J(\theta )=\left(\begin{array}{cccc} \frac{\partial \mu_{1}}{\partial \theta_{1}} & \frac{\partial \mu_{1}}{\partial \theta_{2}} & \ldots & \frac{\partial \mu_{1}}{\partial \theta_{\nu }}\\ \frac{\partial \sigma }{\partial \theta_{1}} & \frac{\partial \sigma }{\partial \theta_{2}} & \ldots & \frac{\partial \sigma }{\partial \theta_{\nu }}\\ \frac{\partial \mu_{2}}{\partial \theta_{1}} & \frac{\partial \mu_{2}}{\partial \theta_{2}} & \ldots & \frac{\partial \mu_{2}}{\partial \theta_{\nu }}\\ \frac{\partial \sigma }{\partial \theta_{1}} & \frac{\partial \sigma }{\partial \theta_{2}} & \ldots & \frac{\partial \sigma }{\partial \theta_{\nu }}\\ \vdots & \vdots & & \vdots \\ \frac{\partial \mu_{j}}{\partial \theta_{1}} & \frac{\partial \mu_{j}}{\partial \theta_{2}} & \ldots & \frac{\partial \mu_{j}}{\partial \theta_{\nu }}\\ \frac{\partial \sigma }{\partial \theta_{1}} & \frac{\partial \sigma }{\partial \theta_{2}} & \ldots & \frac{\partial \sigma }{\partial \theta_{\nu }} \end{array}\right).$$

Noting that we are taking *σ* to be independent of model parameters, all of the partial derivatives of *σ* in (2.6) are zero, except the case where *θ*_*i*_ = *σ*, for some *i* ∈ {1, 2, …, *ν*}, whereby the corresponding partial derivative is unity. Given a set of *N* normally distributed observations at a single point in time, we have an observation process characterized by a mean, *μ*, and standard deviation, *σ*. The Fisher information for such an observation is given by

2.7 $$I(\mu ,\sigma )=\frac{N}{\sigma^{2}}D,\;\text{where }\;D=\mathrm{diag}(1,2).$$

This can be verified by applying equation (2.4) to (1.1). For data at *L* time points with *N*_1_, *N*_2_, …, *N*_*L*_ observations at each time, with constant standard deviation, the Fisher information for the observation process is a 2*L* × 2*L* (block) diagonal matrix,

2.8 $$I(\mu ,\sigma )=\mathrm{diag}\left(\frac{N_{1}}{\sigma^{2}}D,\frac{N_{2}}{\sigma^{2}}D,\ldots ,\frac{N_{L}}{\sigma^{2}}D\right).$$

Similarly, for a model with *M* species, where we have observations of all *M* species at only one time point we recover Fisher information in the form of (2.8). For observations of *M* species at *L* time points we form a 2*LM* × 2*LM* (block) diagonal matrix from (2.8). Assuming a constant standard deviation, for the computations in this work we could more simply express (2.8) as the diagonal matrix $\mathrm{diag}(N_{1}/\sigma^{2},N_{2}/\sigma^{2},\ldots ,N_{L}/\sigma^{2},2\sum N_{i}/\sigma^{2})$, where $\sum N_{i}$ is the total number of observations contributing to our information regarding the standard deviation, and the factor of 2 comes from (2.7). In this case, the model Jacobian as presented in (2.6) is modified such that only the final row includes the partial derivatives with respect to the standard deviation.

Before outlining specific techniques of information geometry, we present a conceptual example to develop some intuition for information geometric concepts. Consider the manifold corresponding to the family of univariate normal distributions parametrized by mean, *μ*, and standard deviation, *σ* > 0. Let $P∼N(\mu_{1},\sigma )$ and $Q∼N(\mu_{2},\sigma )$ be two normal distributions. Geometrically speaking, increasing *σ* reduces the distance between *P* and *Q*; this corresponds to a contraction of the space. Conversely, decreasing the variance dilates the space; as *σ* → 0, the Fisher information, diag(1/*σ*^2^, 1/*σ*^2^), is degenerate and the distance between *P* and *Q* tends to infinity.

Equipped with the Fisher information, we may begin to explain some foundational ideas from information geometry, including geodesic curves, geodesic distances between distributions for statistical models and scalar curvature [49]. We denote the elements of the Fisher information as $I(\theta )=[g_{ij}(\theta )]$, and its inverse $I{\left(\theta \right)}^{-1}=[g^{ij}(\theta )]$, where $\theta =(\theta_{1},\theta_{2},\ldots ,\theta_{\nu })$ are the coordinates of the manifold. While uncertainty in estimates is typically characterized by the Fisher information at only a single point, based on the Cramér–Rao inequality [70], information geometry uses the Fisher information throughout the parameter space. A Riemann geodesic is a curve forming the shortest path between two points in a Riemannian manifold [73]. The length of this shortest curve is referred to as the Fisher or Fisher–Rao distance [74]. We soon discuss a relationship between confidence regions and the length of geodesic curves. Informally, with greater information supporting an MLE, coinciding with an increase in its relative likelihood, confidence regions tighten. This also corresponds to a dilation of the parameter manifold; thereby increasing the geodesic distance between the MLE and other parameter combinations, reflecting their relatively reduced likelihood.

A curve **z**(*s*), parameterized by *s*, connecting the points **z**_1_ = **z**(*s*_1_) and **z**_2_ = **z**(*s*_2_) on a Riemannian manifold, has length [58]

2.9 $$L(z)=\int_{s_{1}}^{s_{2}}\sqrt{\sum_{i,j=1}^{n}\left(g_{ij}(\theta (z(s)))\frac{d\theta_{i}(z(s))}{ds}\frac{d\theta_{j}(z(s))}{ds}\right)}\;ds.$$

A Riemann geodesic is a curve that minimizes *L*(**z**) (2.9), such that the distance between two points on a Riemannian manifold is given by the curve that satisfies

$$d(z_{1},z_{2})=\min\{L(z)\;:\;z(s_{1})=z_{1},z(s_{2})=z_{2}\}.$$

For Gaussian likelihoods, there is an asymptotic relationship between the geodesic distance between the MLE, $\hat{\theta }$, and a point $\theta_{\alpha }$ that corresponds to an *α*-level confidence region on the manifold [75]. The geodesic distance between $\hat{\theta }$ and $\theta_{\alpha }$: $d(\hat{\theta },\theta_{\alpha })$ can be written in terms of the *α*th quantile of the *χ*^2^(*ν*) distribution

2.10 $$d(\hat{\theta },\theta_{\alpha })=\sqrt{\Delta_{\nu ,\alpha }}.$$

Pairing equations (2.3) and (2.10) yields an asymptotic relationship between confidence regions and geodesic length [75]

2.11 $$2(\ell (\hat{\theta })-\ell (\theta ))∼d{\left(\hat{\theta },\theta_{\alpha }\right)}^{2}\;\text{as\textbackslash{} }N\to \infty .$$

In §3, we present likelihood-based confidence regions alongside geodesic curves of the corresponding length, as characterized by (2.10), and comment on the validity of equation (2.11) in a range of scenarios.

Geodesic curves satisfy the following system of differential equations in *n* dimensions [76]:

2.12 $$\frac{d^{2}\theta_{m}}{ds^{2}}+\sum_{i,j=1}^{n}\Gamma_{ij}^{m}\frac{d\theta_{i}}{ds}\frac{d\theta_{j}}{ds}=0,\;m=1,\ldots ,n,$$

where *s* is the parametrization of the geodesic curve, in accordance with equation (2.9), and $\Gamma_{ij}^{m}$ are the Christoffel symbols of the second kind [50], defined as

2.13 $$\Gamma_{ij}^{m}=\frac{1}{2}\sum_{l=1}^{n}g^{ml}\left(\frac{\partial g_{lj}}{\partial \theta_{i}}+\frac{\partial g_{li}}{\partial \theta_{j}}-\frac{\partial g_{ij}}{\partial \theta_{l}}\right).$$

We can convert from Christoffel symbols of the second kind to Christoffel symbols of the first kind by lowering the contravariant (upper) index through multiplication by the metric: $\Gamma_{kij}=g_{km}\Gamma_{ij}^{m}$ [77]. Here, repeated indices, in this case *m*, imply that a summation is to be performed over the repeated index, following the Einstein summation convention [56]. Conversely, we can recover Christoffel symbols of the first kind from Christoffel symbols of the second kind via the inverse metric: $g^{km}\Gamma_{kij}=\Gamma_{ij}^{m}$. Christoffel symbols of the second kind are the connection coefficients of the Levi–Civita connection; the Christoffel symbols are symmetric in the covariant (lower) indices [60]. On an *n*-dimensional manifold, the Christoffel symbol is of dimension *n* × *n* × *n*. Geodesics can be used to construct theoretical confidence regions, to measure the geometric distance between probability distributions and to perform hypothesis testing; for example, to test equality of parameters [48,51,78].

Under certain conditions, analytical expressions can be obtained for the solutions of the geodesic equations, and the corresponding Fisher–Rao distances, for example, in the case of the univariate (1.1) and multivariate (3.2) normal distributions [74,79]. However, we solve equation (2.12) numerically, after converting the second-order ODE to a first-order system of ODEs using standard techniques.

We are also interested in exploring the scalar curvature, also known as the *Ricci scalar*, of our manifolds. To compute the scalar curvature, we must first construct the Riemann tensor, and subsequently the Ricci tensor. As we only require these tensors for computation of the scalar curvature, and do not attempt to interpret these tensors directly in this work, we provide only a limited outline of their interpretation. The Riemann curvature tensor is constructed from the Christoffel symbols and their first partial derivatives. Here, it is convenient to think about these partial derivatives as being with respect to the parameters of interest. Owing to the possibility of raising or lowering indices of Christoffel symbols and tensors via the metric, there are several equivalent expressions for computing the Riemann curvature tensor [77]. The elements of the Riemann tensor of the first kind can be written as

2.14 $$R_{ijkl}=\frac{\partial \Gamma_{\;jli}}{\partial k}-\frac{\partial \Gamma_{\;jki}}{\partial l}+\Gamma_{ilr}\Gamma_{\;jk}^{\;r}-\Gamma_{ikr}\Gamma_{\;jl}^{\;r}.$$

The Riemann tensor of the first kind is a (0, 4) tensor (with no contravariant indices and four covariant indices), and can be converted to the (1, 3) Riemann tensor of the second kind via the inverse of the metric: $g^{im}R_{ijkl}=R_{\;jkl}^{m}$. On an *n*-dimensional manifold, the Riemann tensor is of dimension *n* × *n* × *n* × *n*; owing to various symmetries, however, there are far fewer independent elements [80]. The Riemann tensor provides information about the intrinsic curvature of the manifold. A geometric interpretation is that a vector from a point on the manifold, parallel transported around a parallelogram, will be identical to its original value when it returns to its starting point if the manifold is flat. In this case, the Riemann tensor vanishes. If the manifold is not flat, the Riemann tensor can be used to quantify how the vector differs following this parallel transport [81].

From the Riemann tensor of the second kind, we can compute the Ricci tensor of the first kind. The Ricci tensor, *R*_*ij*_, is obtained by contracting the contravariant index with the third covariant index of the Riemann tensor of the second kind; that is,

2.15 $$R_{ij}=R_{ijm}^{m}.$$

On an *n*-dimensional manifold, the Ricci tensor is of dimension *n* × *n* and is symmetric [81]. The Ricci tensor can quantify the changes to a volume element as it moves through the manifold, relative to Euclidean space [81].

The scalar curvature, Sc, can be obtained as a contraction of the Ricci tensor

2.16 $$\mathrm{Sc}=g^{ij}R_{ij}.$$

The scalar curvature is invariant; it does not change under a change of coordinates (re-parametrization). For Gaussian likelihoods, the corresponding manifold is flat, characterized by zero scalar curvature everywhere. As such, the scalar curvature provides a measure of how the likelihood of the underlying statistical model deviates from being Gaussian—often referred to as *non-Gaussianity* in the physics and cosmology literature—irrespective of the parametrization [60]. As we will explore in §3, it can also provide insights into parameter identifiability.

### 2.3. Hypothesis testing

Here we outline the approach for performing likelihood-ratio-based hypothesis tests, and hypothesis tests based on geodesic distance. As we consider synthetic data in this work, we know the *true* parameter values, ***θ***_*t*_. In practical applications this is not the case. As such, we may seek to test whether some previously held notion about the true parameters, ***θ***_*t*_ = ***θ***_0_, is supported by the data, based on the computed MLE. This could be investigated via the following hypothesis test:

2.17 $$\begin{array}{cc} & H_{0}\;:\;\theta_{t}=\theta_{0}\\ \;\mathrm{and}\; & H_{1}\;:\;\theta_{t}\neq \theta_{0}. \end{array}\}$$

From equation (2.3), the test statistic for such a likelihood-ratio-based hypothesis test can be expressed as

2.18 $$\lambda_{\mathrm{LR}}=-2(\ell (\theta_{0})-\ell (\hat{\theta })),$$

where asymptotically as *N* → ∞, *λ*_LR_ ∼ *χ*^2^(*ν*), following Wilk’s theorem [36]. From the asymptotic relationship given in equation (2.11), it follows that under the same asymptotic relationship the test statistic for a hypothesis test based on geodesic distance is [78]

2.19 $$\lambda_{\mathrm{GD}}=d{\left(\theta_{0},\hat{\theta }\right)}^{2}.$$

The likelihood values required to compute equation (2.18) can be obtained directly by evaluating equation (2.1). To compute the geodesic distance between two specific points in parameter space, as required by equation (2.19), it is necessary to solve a boundary value problem to obtain the geodesic curve between ***θ***_0_ and $\;\hat{\theta }$. Approximate *p*-values can be computed from these test statistics as $1-F_{\chi^{2}(\nu )}(\lambda_{\mathrm{LR}})$ and $1-F_{\chi^{2}(\nu )}(\lambda_{\mathrm{GD}})$, respectively, where $F_{\chi^{2}(\nu )}$ is the cumulative distribution function of *χ*^2^(*ν*) [1]. We provide practical examples of each of these approaches to hypothesis testing in §3.

### 2.4. Numerical implementation

All numerical techniques used to produce the results in this work are implemented in the open source Julia language [69]; we use a combination of existing Julia packages and bespoke implementations. There are several aspects of numerical computation in this work, including approximate solutions to systems of ODEs, differentiation with both finite differences and forward mode automatic differentiation, likelihood computation and nonlinear optimization. Nonlinear optimization for obtaining MLEs and parameter combinations corresponding to particular confidence levels is performed with the Julia package NLopt.jl, using the Bound Optimization by Quadratic Approximation (BOBYQA) algorithm. BOBYQA is a derivative-free algorithm for solving bound constrained optimization problems [82]. Approximate solutions to ODEs are obtained using the Julia package DifferentialEquations.jl [83]. The second-order Heun’s method [84], a two-stage Runge–Kutta method, is used for obtaining contours of the log-likelihood function to form approximate likelihood-based confidence regions [1]. Heun’s method is implemented as Heun() in DifferentialEquations.jl. Approximate solutions to geodesic differential equations are obtained using the Tsitouras implementation of the Runge–Kutta method, which employs Runge–Kutta pairs of orders 5 and 4 [85], implemented as Tsit5() in DifferentialEquations.jl. Boundary value problems for geodesic-distance-based hypothesis tests are solved using the DifferentialEquations.jl implementation of a shooting method, using Tsit5(). Code for reproducing all examples in this work is available on GitHub.

## 3. Results

In this section, we present results combining likelihood-based parameter inference and uncertainty quantification with ideas from information geometry, including geodesic curves and scalar curvature. We apply these techniques to univariate and multivariate normal distributions, linear, exponential and logistic population growth models and the SIR model. Through these canonical examples, we explore pedagogically differences in the inference and information geometry results that arise as we consider parameter estimation and uncertainty for increasingly complex systems.

Synthetic data for the univariate and multivariate normal distributions are generated by sampling from the respective distributions given in equation (3.1). For simplicity, in this work we consider synthetic data from uncorrelated observation processes with constant standard deviation in both time and parameter space. However, we note that the techniques presented in this work can be generalized to handle data with non-constant variance and for other distributions where the Fisher information is available [72].

3.1 $$\begin{array}{rl} \mathrm{Univariate}\; & :\;x_{i}∼N(\mu ,\sigma^{2}),\\ \mathrm{Multivariate}\; & :\;x_{i}∼\mathrm{MVN}(\mu ,\Sigma ), \end{array}$$

where $\Sigma =\mathrm{diag}(\sigma^{2})$ is the covariance matrix. For the population growth and SIR models considered in this work, synthetic data are generated by drawing from a normal distribution with mean described by the model solution and a prescribed standard deviation, effectively substituting *μ* = *μ*(***θ***, *t*) in equation (3.1) for observation processes with a single variable and ***μ*** = ***μ***(***θ***, *t*) for observation processes with several variables. When *σ* is one of the parameters to be estimated, *σ* ∈ ***θ***, but ***μ*** does not depend on *σ*. Parameter values that we use to generate synthetic data correspond to parameter estimates inferred from field data in the literature [2,16].

We present a series of figures in this section visualizing the normalized log-likelihood, $\hat{\ell }$, and scalar curvature, Sc, as heatmaps, with likelihood-based 95% confidence regions and geodesics with a length corresponding to a 95% confidence distance superimposed. All results are computed numerically, as outlined in §2, with code available on GitHub. Unless otherwise indicated, each set of geodesics includes 20 geodesics with initial velocities corresponding to equidistant points uniformly distributed on the circumference of a unit circle. As such, the apparent clustering of geodesics in some examples highlights differences in the scaling and stretching of parameter spaces. Each scalar curvature and log-likelihood heatmap is computed on a uniformly discretized 100 × 100 grid.

### 3.1. Normal distributions

**Figure 1.**
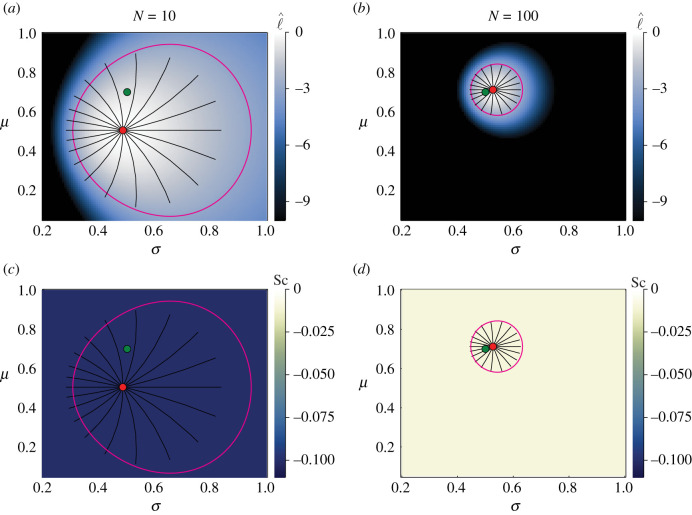
Univariate normal distribution with inferred mean, *μ*, and standard deviation, *σ*. Heatmaps visualize the normalized log-likelihood, $\hat{\ell }$ (*a*,*b*), and the scalar curvature, Sc (*c*,*d*). True parameter values, (*μ*, *σ*) = (0.7, 0.5), are marked with green discs, with the MLEs indicated using red discs. Magenta curves correspond to likelihood-based 95% confidence regions. Black lines are geodesic curves emanating from the MLEs, with a geodesic length corresponding to a theoretical 95% confidence region. Increasing the number of data points, *N*, tightens the confidence regions, improves the correspondence between geodesic curves and likelihood-based confidence regions and reduces the scalar curvature.

We first consider parameter inference and information geometry techniques applied to observations drawn directly from univariate and bivariate normal distributions, with no underlying process model. In figure 1, we present results for the univariate normal distribution (1.1), estimating ***θ*** = (*μ*, *σ*). The true mean and standard deviation used to generate data are (*μ*, *σ*) = (0.7, 0.5). Estimates are obtained via maximum likelihood estimation. MLEs of normal variance are known to provide biased underestimates [36], and the derivation of the Fisher information assumes an unbiased estimator [86]. This may partially explain the particular differences observed between the likelihood-based confidence region and the endpoints of the geodesics in figure 1, wherein the geodesics not only appear to suggest a tighter confidence region but also appear to be biased towards parameter space with smaller standard deviation. As the number of observations increases from *N* = 10 to *N* = 100, we observe not only that the MLE more precisely estimates the true parameter values, but also that the endpoints of the geodesic curves more closely correspond to the likelihood-based confidence regions. This is consistent with both the theoretical asymptotic relationship between geodesic length and likelihood-based confidence regions given in equation (2.11), and also the bias of the MLE for standard deviation decreasing, as *N* increases.

The manifold representing the family of normal distributions parametrized by ***θ*** = (*μ*, *σ*) has constant scalar curvature Sc = −1. Owing to the additive nature of the Fisher information, having *N* observations results in a constant scalar curvature of Sc = −1/*N*, as presented in figure 1*c*,*d*. It is straightforward, although tedious, to verify this result through combining equations (1.4), (2.13)–(2.16).

The probability density function for the multivariate normal distribution with two independent variables, x,y∈R, with constant standard deviation *σ* is

3.2 $$p(x,y;\mu_{1},\mu_{2},\sigma )=\frac{1}{2\pi \sigma^{2}}\exp\left(-\left(\frac{{\left(x-\mu_{1}\right)}^{2}+{\left(y-\mu_{2}\right)}^{2}}{2\sigma^{2}}\right)\right).$$

**Figure 2.**
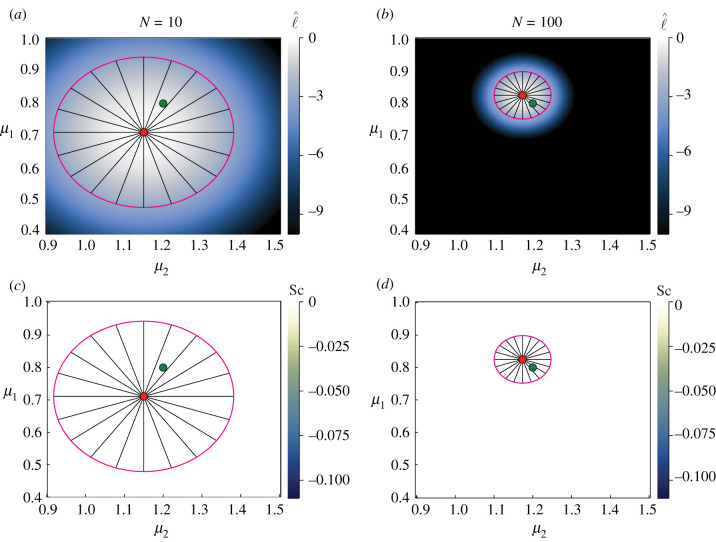
Multivariate normal distribution with inferred means, *μ*_1_ and *μ*_2_, with known constant standard deviation, *σ* = 0.3. Heatmaps visualize the normalized log-likelihood, $\hat{\ell }$ (*a*,*b*), and the scalar curvature, Sc (*c*,*d*). True parameter values, (*μ*_1_, *μ*_2_) = (0.8, 1.2), are marked with green discs, with the MLEs indicated using red discs. Magenta curves correspond to likelihood-based 95% confidence regions. Black lines are geodesic curves emanating from the MLEs, with geodesic lengths corresponding to a theoretical 95% confidence region. Increasing the number of data points, *N*, tightens the confidence regions. In contrast to the univariate case where we infer standard deviation in figure 1, when only inferring the mean parameters of the multivariate normal distribution, we see that even with few observations, *N* = 10, the geodesics and likelihood-based confidence regions match closely. As we are estimating means only, and there is no model-induced curvature, the scalar curvature is zero everywhere.

In equation (3.2), there are three parameters that we could estimate from data: $\Theta =(\mu_{1},\mu_{2},\sigma )$. As we estimated the mean and standard deviation for the univariate normal distribution in figure 1, we consider inference of both means for the multivariate normal, ***θ*** = (*μ*_1_, *μ*_2_). Results are presented in figure 2. Even with a small number of observations (*N* = 10), we observe an excellent match between the likelihood-based confidence regions and geodesics when only estimating means. As expected, increasing *N* results in an MLE closer to the true values, and tighter confidence regions. We also observe that the confidence regions are symmetric with respect to each mean parameter. When estimating only the mean parameters of the multivariate normal distribution, we see that the scalar curvature is zero everywhere. This is to be expected, as the Fisher information for normally distributed observation processes, equation (2.7), depends only on the standard deviation and not the mean. As such all of the partial derivatives used to construct the Christoffel symbols (2.13) are zero; this vanishing of the Christoffel symbols translates to zero scalar curvature through equations (2.14)–(2.16). We also observe that, in contrast to the evident curvature of the geodesics for the univariate normal case presented in figure 1, the geodesic curves in figure 2 appear perfectly straight when plotted in Euclidean geometry. The Riemann tensor (2.14) is zero everywhere when inferring multivariate normal means. This suggests that the manifold is flat.

**Figure 3.**
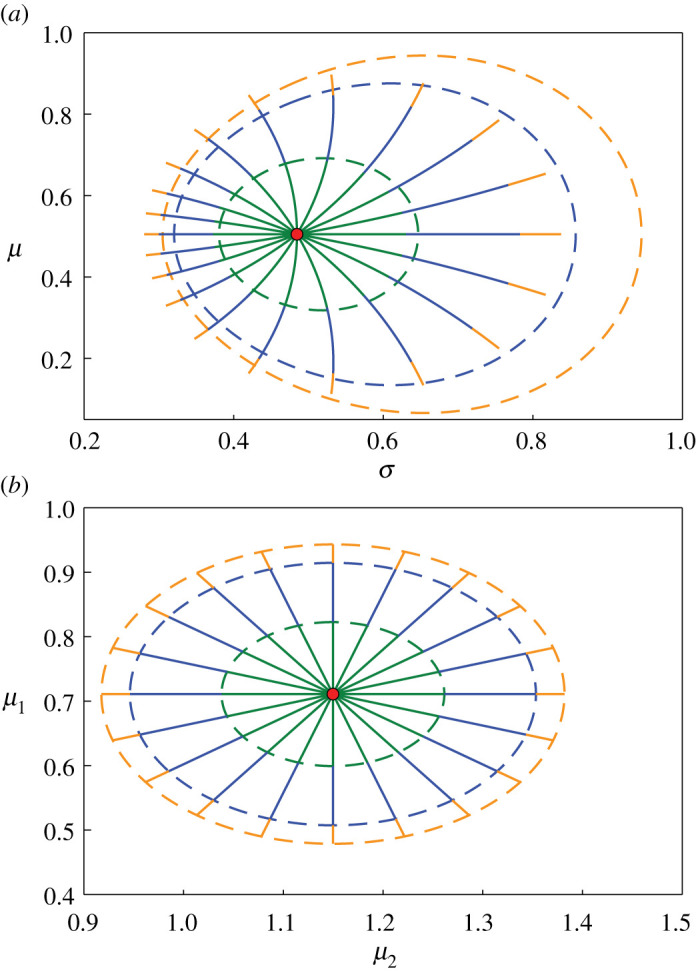
Comparison of confidence regions at intermediate-likelihood values and geodesic distances. Results correspond to (*a*) univariate normal distribution with inferred mean, *μ*, and standard deviation, *σ*, as considered in figure 1*a*, and (*b*) multivariate normal distribution with inferred means, *μ*_1_ and *μ*_2_, as considered in figure 2*a*. MLEs are indicated using red discs. Dashed curves correspond to likelihood-based 50% (green), 90% (blue) and 95% (orange) confidence regions. Solid lines are geodesic curves emanating from the MLEs, with geodesic lengths within a theoretical 50% (green), 90% (blue) and 95% (orange) confidence distance.

Results presented in this work predominantly feature 95% confidence regions. We note that, although this choice is common [87], it is also arbitrary, and equivalent analysis could be performed at different confidence levels. In examples where the geodesic endpoints approximately align with the likelihood-based confidence regions at the 95% level, we expect intermediate points along the geodesics to also approximately align with corresponding likelihood contours, in accordance with equation (2.11). However, in examples where we observe a mismatch between geodesic endpoints and likelihood-based confidence intervals at the 95% level, we do not expect intermediate points along geodesics to correspond to likelihood contours. This is demonstrated in figure 3.

Having considered the techniques as applied directly to distributions, we now incorporate ODE-based process models, such that our observations are normally distributed about the solution of a mathematical model.

### 3.2. Population growth models

**Figure 4.**
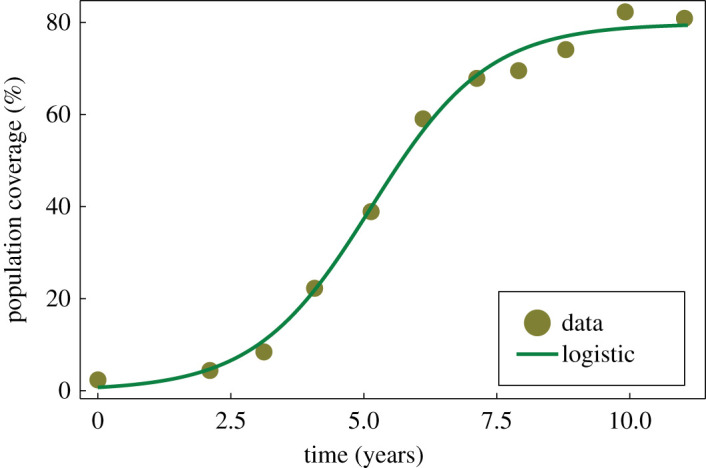
Markers correspond to data from field studies, representing the percentage of area in a region covered by hard corals, as the coral population regrows following depletion by an external event [16]. Data originally extracted from the Australian Institute of Marine Science (AIMS) Long Term Monitoring Program (LTMP) eAtlas (eatlas.org.au/gbr/ltmp-data). A logistic model is fitted to the data in [16], with inferred parameters: *r* = 0.9131 (yr^−1^), C(0)=0.7237%, K=79.74% and standard deviation *σ* = 2.301; this is reproduced here as the green curve.

The canonical logistic growth model, alongside generalizations and related sigmoid models such as Gompertz and Richards’ models, have been extensively applied to study population growth dynamics in the life sciences [16,88]. In figure 4, we present data from the literature describing the area covered by hard corals in a region as they regrow following an adverse event. This can be modelled as a logistic growth process [16]. Logistic growth of a population with density *C*(*t*) is characterized by a growth rate *r* > 0, initial condition *C*(0) > 0 and carrying capacity *K* > 0. Treating parameter values $\left(r,C(0),K)=(0.9131\;({\mathrm{yr}}^{-1}),0.7237\text{\%},79.74\text{\%}\right)$, and standard deviation σ=2.301%, inferred in the literature from this field data as the *true* values, we generate various synthetic datasets with multiple observations at various time points.

**Figure 5.**
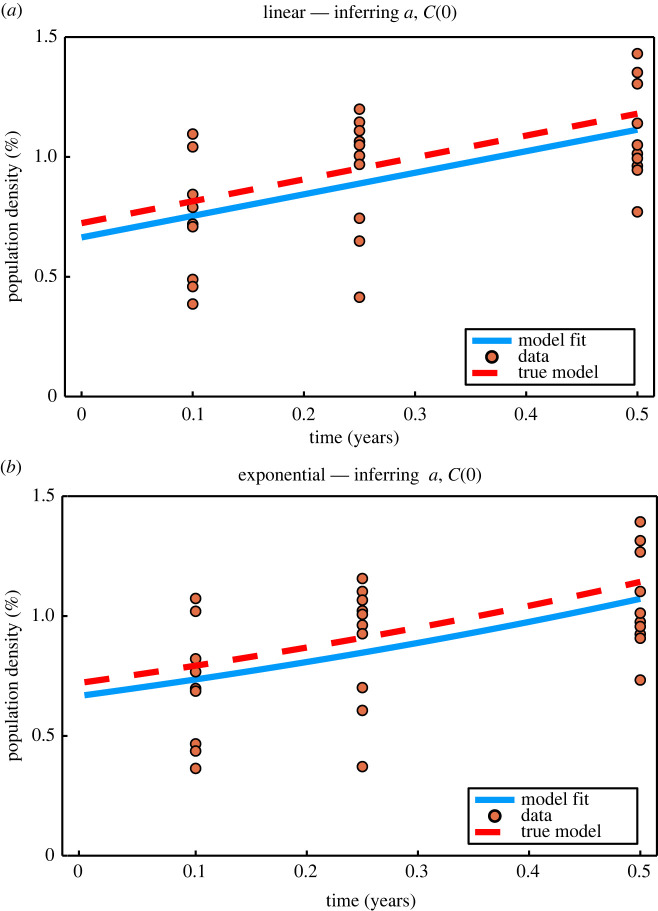
Example synthetic data generated from the linear and exponential models with comparison of early time linear and exponential model fits, inferring *a* and *C*(0). *N* = 10 observations per time point, with time points *T* = (0.1, 0.25, 0.5). True parameter values are *a* = 0.9131, *C*(0) = 0.7237, with known standard deviation, *σ* = 0.2301. For generating synthetic early time linear and exponential data, we reduce the standard deviation relative to the *σ* = 2.301 computed from the logistic model, as early time data produced with *C*(0) = 0.7237 and *σ* = 2.301 produce negative population density observations. Inference produces MLEs of $\left(\hat{a},\hat{C(0)})=(0.8988,0.6642\right)$ for the linear model and $\left(\hat{a},\hat{C(0)})=(0.9412,0.6695\right)$ for the exponential model.

**Figure 6.**
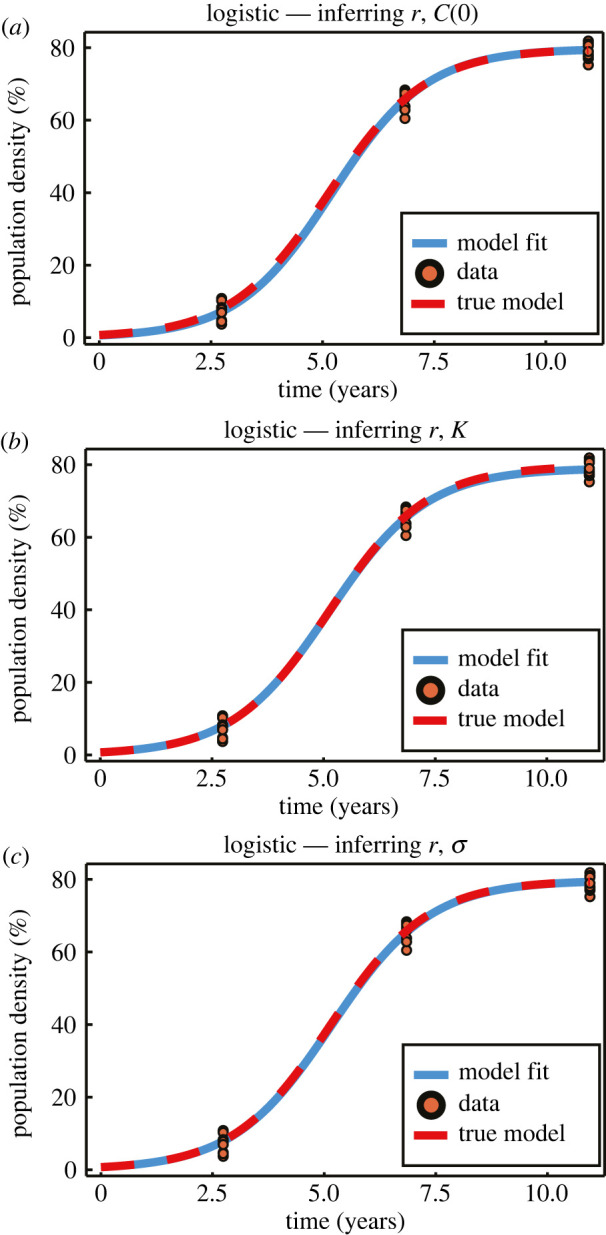
Example synthetic data generated from the logistic growth model. The logistic model is fitted to the synthetic data, inferring pairwise combinations of *r* with *C*(0), *K* and *σ*. Observations are made at *T* = (2.74, 6.84, 10.95) years, with *N* = 10 observations per time point. True parameter values are *r* = 0.9131, *C*(0) = 0.7237, *K* = 79.74 and *σ* = 2.301.

The logistic growth model is well approximated by the exponential growth model when *C*(*t*) ≪ *K* [89], and early time exponential growth is approximately linear. Before considering the inference and information geometry techniques as applied to the logistic model, we first consider the more fundamental linear and exponential growth models. In figure 5, we present example synthetic linear and exponential data, and in figure 6 synthetic logistic data. In the context of population growth models, the presence of variability in observations at a single time point could reflect, for example, measurement error, variability in population estimates or expert judgement [90].

#### 3.2.1. Linear growth

Linear growth describes growth at a constant rate, independent of the population density. The linear growth model and solution are given by

$$\frac{dC}{dt}=a\;\mathrm{and}\;C(t)=at+C(0).$$

**Figure 7.**
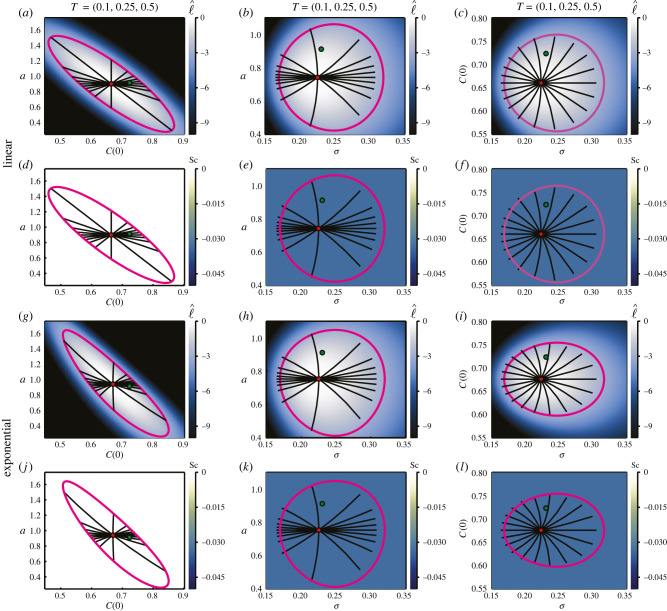
Linear (*a*–*f*) and exponential (*g*–*l*) models with inferred pairwise combinations of growth rate, *a*, initial condition, *C*(0), and standard deviation, *σ*. Heatmaps visualize the normalized log-likelihood, $\hat{\ell }$ (*a*–*c*, *g*–*i*), and the scalar curvature, Sc (*d*–*f*, *j*–*l*). Observations are made at *T* = (0.1, 0.25, 0.5), with 10 observations per time point, corresponding to the example data presented in figure 5. The true parameter values are marked with green discs, with the MLEs indicated using red discs. Magenta curves correspond to likelihood-based 95% confidence regions. Black lines are geodesic curves emanating from the MLEs, with lengths corresponding to a theoretical 95% confidence distance. True values of model parameters correspond to the logistic growth parameters; *a* = 0.9131, *C*(0) = 0.7237, with reduced standard deviation *σ* = 0.2301.

With parameters Θ=(a,C(0),σ), μ(Θ,t)=at+C(0) describes the expected model behaviour. In figure 7*a*–*f*, we present inference results for the linear model for all pairwise combinations of Θ. The partial derivatives of the linear model with respect to the parameters *a* and *C*(0), required to form the Jacobians, **J**(***θ***), are

$$\frac{\partial \mu (\Theta ,t)}{\partial a}=t\;\mathrm{and}\;\frac{\partial \mu (\Theta ,t)}{\partial C(0)}=1.$$

Recall from equation (2.6) that we only require the partial derivatives corresponding to unknown parameters in any given example. When estimating ***θ*** = (*a*, *C*(0)) we find that, similar to the multivariate normal case where we estimate means, the scalar curvature is zero everywhere. We also observe that the endpoints of the geodesics align with the likelihood-based confidence region. We stress that this arises through the relationship in equation (2.11), and is not forced to occur via termination of the numerical solution of the ODE once it reaches the likelihood-based confidence region. However, due to the relationship between *a* and *C*(0), we find that the confidence regions in this case are not symmetric about the MLE with respect to each parameter. Rather, we see that for a given normalized log-likelihood value a larger growth rate corresponds to a smaller initial condition, and vice versa. This aligns with our intuition when considering fitting a straight line through data, as presented in figure 5*a*; lines with a greater slope (*a*) must start lower (*C*(0)) to fit the data.

When one of the parameters to be estimated is *σ*, we observe similar results to the univariate normal case; geodesic endpoints are offset in the direction of decreasing *σ* relative to the likelihood-based confidence regions, and there is constant scalar curvature of Sc = −1/*N*. The geodesics and confidence regions appear symmetric with respect to the model parameter, about the MLE.

#### 3.2.2. Exponential growth

Exponential growth describes growth at a rate proportional to the size of the population. The exponential growth model and solution are

$$\frac{dC}{dt}=aC\;\mathrm{and}\;C(t)=C(0)\exp(at).$$

With parameters Θ=(a,C(0),σ), μ(Θ,t)=C(0)exp⁡(at) describes the expected model behaviour. The partial derivatives of the exponential model with respect to the parameters *a* and *C*(0), required to form the Jacobians, **J**(***θ***), are

$$\frac{\partial \mu (\Theta ,t)}{\partial a}=tC(0)\exp(at)\;\mathrm{and}\;\frac{\partial \mu (\Theta ,t)}{\partial C(0)}=\exp(at).$$

By construction, as detailed in figure 5, the linear and exponential models with identical parameters and initial conditions produce very similar behaviours over a sufficiently small time scale. This is seen when comparing the inference results for the exponential model, presented in figure 7*g*–*l*, with the corresponding linear results in figure 7*a*–*f*. When inferring ***θ*** = (*a*, *σ*), deviations from the corresponding linear results are minimal. The likelihood-based confidence region and corresponding geodesic endpoints for ***θ*** = (*a*, *C*(0)) are marginally tighter and less elliptical. When inferring ***θ*** = (*C*(0), *σ*), we find that the confidence region for the exponential model is narrower with respect to *C*(0) than that of the linear model, though near-identical with respect to *σ*. As for the linear case, the scalar curvature is Sc = −1/*N* everywhere when *σ* is one of the unknown parameters, and zero everywhere otherwise.

#### 3.2.3. Logistic growth

Logistic growth describes growth at a rate dependent on the size of the population, with growth ceasing once the population reaches a carrying capacity. For sufficiently small populations relative to the carrying capacity, logistic growth is approximately exponential [89]. As the population approaches the carrying capacity, the rate of growth slows. The logistic growth model is

$$\frac{dC(t)}{dt}=rC(t)\left(1-\frac{C(t)}{K}\right),$$

with solution

3.3 $$C(t)=\frac{C(0)K}{C(0)+(K-C(0))\exp(-rt)}.$$

The long-time limit of equation (3.3) is lim _*t*→∞_*C*(*t*) = *K*. The behaviour of the logistic model can be described by the three model parameters and standard deviation: Θ=(r,C(0),K,σ). We can compute the partial derivatives required to form the Jacobian matrices, **J**(***θ***), analytically,

3.4 $$\begin{array}{rl} \mu (\Theta ,t) & =C(r,C(0),K,t)=\frac{C(0)K}{C(0)+(K-C(0))\exp(-rt)},\\ \frac{\partial \mu (\Theta ,t)}{\partial r} & =\frac{C(0)Kt(K-C(0))\exp(-rt)}{{\left((K-C(0))\exp(-rt)+C(0)\right)}^{2}},\\ \frac{\partial \mu (\Theta ,t)}{\partial C(0)} & =\frac{K^{2}\exp(rt)}{{\left(C(0)(\exp(rt)-1)+K\right)}^{2}}\\ \mathrm{and}\;\frac{\partial \mu (\Theta ,t)}{\partial K} & =\frac{C{\left(0\right)}^{2}\exp(rt)(\exp(rt)-1)}{{\left(C(0)(\exp(rt)-1)+K\right)}^{2}}. \end{array}\}$$

Recall that ***θ*** includes only the unknown parameters to be estimated, so the components required from equation (3.4) to form **J**(***θ***) depend on the specific example.

Example synthetic logistic data are presented in figure 6, demonstrating the model fits for ***θ*** = (*r*, *C*(0)), ***θ*** = (*r*, *K*) and ***θ*** = (*r*, *σ*). With data at *early*, *mid-* and *late* time, *T* = (*t*_1_, *t*_2_, *t*_3_) = (2.74, 6.84, 10.95) yr, we observe an excellent model fit in all cases. The fit is best when ***θ*** = (*r*, *σ*), as only one model parameter is unknown. Comparing ***θ*** = (*r*, *C*(0)) and ***θ*** = (*r*, *K*) we observe a marginally better fit at late time when *K* is known, and at early time when *C* is known, as expected.

**Figure 8.**
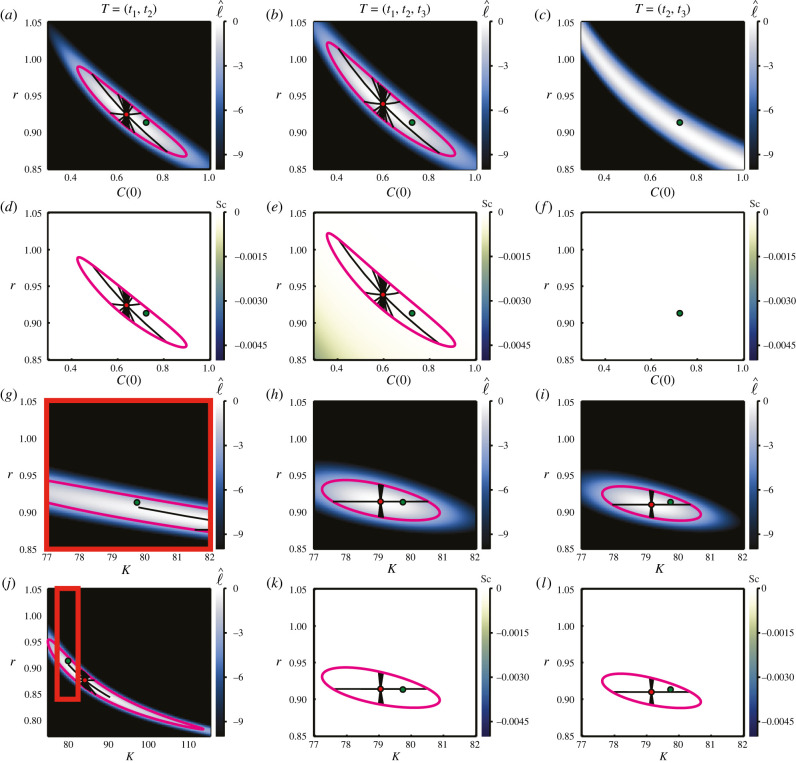
Logistic growth model with inferred growth rate, *r*, and initial condition, *C*(0) (*a*–*f*), and with inferred growth rate, *r*, and carrying capacity, *K* (*g*–*l*). True parameters are as noted in figure 6, with known standard deviation, *σ* = 2.301. Heatmaps visualize the normalized log-likelihood (*a*–*c*, *g*–*j*) and the scalar curvature (*d*–*f*, *k*–*l*). The true parameter values are marked with green discs, with the MLEs indicated using red discs. Magenta curves correspond to likelihood-based 95% confidence regions. Black lines are geodesic curves emanating from the MLEs, with lengths corresponding to a theoretical 95% confidence distance. Columns of the figure correspond to observations from early–mid time (*T* = (*t*_1_, *t*_2_)), early–mid–late time (*T* = (*t*_1_, *t*_2_, *t*_3_)) and mid–late time (*T* = (*t*_2_, *t*_3_)), where (*t*_1_, *t*_2_, *t*_3_) = (2.74, 6.84, 10.95) yr. Each plot reflects a total of 30 observations, distributed equally between the specified time points. The red outline in (*j*) corresponds to the (zoomed in) region (*g*), also outlined in red. In (*g*,*j*), we plot 1000 geodesics to observe the geodesic near the true parameter values. We do not present Sc corresponding to (*g*,*j*); however, it is zero everywhere.

We present inference results for the logistic model for ***θ*** = (*r*, *C*(0)) in figure 8*a*–*f* and for ***θ*** = (*r*, *K*) in figure 8*g*–*l*. We do not present further results of inferring *σ* for the logistic model, as little insight is gained beyond what we glean from the linear and exponential growth results. For ***θ*** = (*r*, *C*(0)), the normalized log-likelihood reflects the same relationship between growth rate and initial condition as for the linear and exponential cases. With early–mid time data and early–mid–late time data, we are able to infer ***θ*** = (*r*, *C*(0)). With only mid–late time data, we find that the parameters are not practically identifiable. This can be seen from figure 8*c*; the normalized log-likelihood remains above the threshold prescribed in equation (2.3), and a closed likelihood-based 95% confidence region cannot be constructed. This is also reflected in figure 8*f* alongside zero scalar curvature, such that the plot appears empty. Comparing figure 8*a*,*b*, and noting that they each rely on the same total number of observations, the importance of early and mid-time data when inferring ***θ*** = (*r*, *C*(0)) is reinforced. The confidence region is tighter with only early–mid data than with the same amount of data spread across early, mid- and late times.

Inferring ***θ*** = (*r*, *K*) reflects similar behaviour. In figure 8*j* and the associated zoomed-in view (figure 8*g*), inferring the carrying capacity from only early–mid time data results in an extremely wide confidence region, though the parameters remain identifiable. The geodesics emanating from the MLE match the likelihood-based confidence region very well in directions where the normalized log-likelihood is steep; however, they do not quite reach the true parameter value in the direction where the normalized log-likelihood is relatively flat. Comparing figure 8*g*,*j* with figure 8*h*,*i*, the MLE for ***θ*** = (*r*, *K*) appears to be relatively poor when only early–mid time data are used.

When considering ***θ*** = (*r*, *C*(0)), we see that, with early–mid time data and mid–late time data, the scalar curvature is zero everywhere. However, introducing a third time point (early–mid–late data) results in a non-constant negative scalar curvature. We expect that this relates to the relationships between the parameters, and the difference between a mapping (where we have two pieces of information and two parameters to estimate) and a fit (where we have three pieces of information and two parameters to estimate). We do not observe similar behaviour for ***θ*** = (*r*, *K*) with data at three time points; the scalar curvature still appears to be zero everywhere. One explanation for this is that data at *t*_1_, where *C*(*t*) ≪ *K*, may be effectively independent of *K*, providing no information about *K* [15]. This may effectively reduce the problem to a mapping. Given that the scalar curvature is a feature of the manifold rather than the data, it is of interest to investigate what would happen were the true parameters to lie within this region of non-constant scalar curvature.

**Figure 9.**
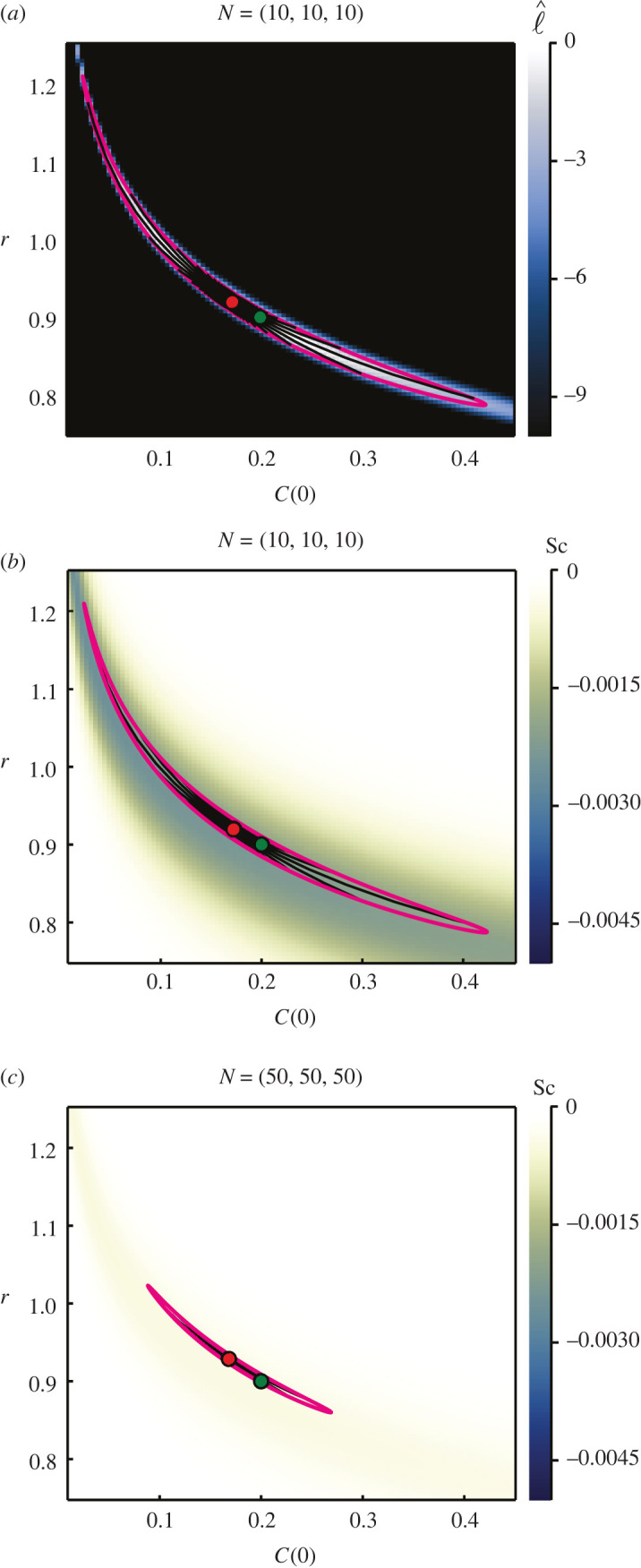
Logistic growth model with inferred growth rate, *r*, and initial condition, *C*(0), with known standard deviation, *σ* = 2.301, and carrying capacity, *K* = 79.74. Heatmaps visualize the normalized log-likelihood (*a*) and the scalar curvature (*b*,*c*). Data are observed at *T* = (2.74, 6.84, 10.95), with 10 (*a*,*b*) and 50 (*c*) observations per time point. The true parameter values are marked with green discs, with MLEs indicated using red discs. Magenta curves correspond to likelihood-based 95% confidence regions. Black lines are 100 geodesic curves emanating from the MLEs, with lengths corresponding to a theoretical 95% confidence distance.

To address this, we generate an alternate set of synthetic logistic growth data using parameter values from within the high curvature region, (*r*, *C*(0)) = (0.9, 0.2), with (*K*, *σ*) = (79.74, 2.301) as before. Inference results are presented in figure 9. We still observe correspondence between the endpoints of the geodesics and the likelihood-based confidence region; however, the confidence region is now significantly narrower and reflects a more hyperbolic shaped relationship between *r* and *C*(0) in terms of the normalized log-likelihood. Increasing the number of observations, as depicted in figure 9*c*, has the expected effects of tightening the confidence region and reducing the scalar curvature. This reduces the apparent curvature of the confidence region.

### 3.3. SIR epidemic model

The SIR model describes the dynamics of epidemic transmission through a population [2]. Populations are assumed to be composed of susceptible, *s*(*t*), infected, *i*(*t*), and recovered, *r*(*t*), individuals. The total population, N, is held constant. When analysing the SIR model in this work, we consider each population as a proportion of the total population, such that S(t)=s(t)/N, I(t)=i(t)/N and R(t)=r(t)/N. Quantities N, *s*(*t*), *i*(*t*) and *r*(*t*) are dimensional with dimensions of number of individuals, whereas *S*(*t*) ∈ [0, 1], *I*(*t*) ∈ [0, 1] and *R*(*t*) ∈ [0, 1] are dimensionless quantities with the property that *S*(*t*) + *I*(*t*) + *R*(*t*) = 1. While the coral re-growth process considered in the population model examples takes place over many years, epidemics occur over a time scale of days or weeks. As such, we now take *t* to represent time as measured in days, rather than years. The parameters of the SIR model are the infection rate, *β* (d^−1^), and the rate at which infected individuals are removed, *γ* (d^−1^), for example, via recovery from the infection

3.5 $$\begin{array}{rl} \frac{dS}{dt} & =-\beta SI,\\ \frac{dI}{dt} & =\beta SI-\gamma I\\ \;\mathrm{and}\;\frac{dR}{dt} & =\gamma I. \end{array}\}$$

Alongside *β* and *γ* we could also treat the initial conditions, *S*(0), *I*(0) and *R*(0), as unknown parameters to be estimated. The standard SIR model presented in equation (3.5) is sufficient for our purposes in this work; however, numerous extensions to the SIR model are considered in the literature. These extensions incorporate factors such as age structure, birth and death, exposed but not yet infected individuals, seasonality, competition between infectious strains, waning immunity, vaccination and spatial structure [2,91–93].

**Figure 10.**
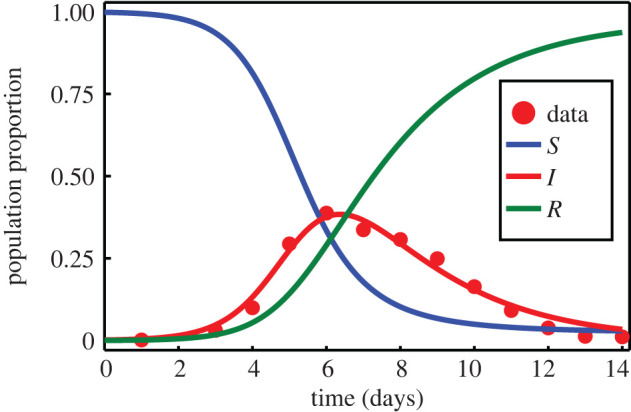
Data marked with red discs represent the number of infected individuals during an influenza outbreak in a boarding school [2]. Susceptible, *S*(*t*), infected, *I*(*t*), and recovered, *R*(*t*), populations are modelled according to equation (3.5) based on parameters inferred in [2], *β* = 1.6633, *γ* = 0.44036; we treat these as the true parameters when generating synthetic data. Initial population proportions are *S*(0) = 762/763, *I*(0) = 1/763 and *R*(0) = 0.

**Figure 11.**
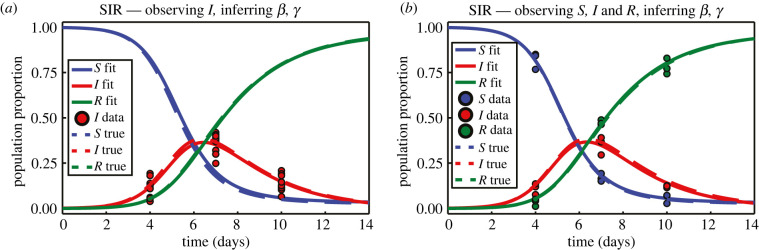
Example synthetic data generated from the SIR model under the scenarios where: (*a*) only the number of infected individuals is observed and (*b*) we have observations pertaining to all three populations. Observations are marked with discs. Populations are modelled according to equation (3.5) based on parameters inferred in [2]. Initial population proportions are *S*(0) = 762/763, *I*(0) = 1/763 and *R*(0) = 0. In (*a*) there are *N* = 10 observations at each time point, and we prescribe *σ* = 0.05; in (*b*) there are three observations per time point, per population, with prescribed *σ* = 0.03. The choices of *σ* are sufficiently small that the data generated consist only of positive observed population proportions.

Data pertaining to the proportion of a population infected during an influenza outbreak in a boarding school are presented in figure 10. Observations in the original data record the number of infected individuals over a 14-day period [2], in a population of N=763, with initial populations (*s*(0), *i*(0), *r*(0)) = (762, 1, 0). These data are used in [2] to estimate parameters for the SIR model, which, after scaling such that *S* + *I* + *R* = 1, are *β* = 1.6633 and *γ* = 0.44036. We treat these values as the true parameters when generating synthetic data, examples of which are presented in figure 11. In the context of an SIR model, the presence of multiple observations at a single time point could reflect, for example, reporting errors, uncertainty in test accuracy or expert judgement [94,95]. In the boarding school data considered in [2], observations pertain only to the number of infected individuals. Given that the SIR model features multiple populations, data could in theory contain observations of the other populations also. Example synthetic data with observations on all three populations are presented in figure 11*b*.

The SIR model as described in equation (3.5) does not admit a closed form analytical solution, so we apply numerical techniques to solve the system. This becomes somewhat computationally expensive, as the Fisher information computations rely on partial derivatives of the model solution with respect to the parameters to form the model Jacobian, and the information geometry computations require partial derivatives of the Fisher information up to second order. Approximating these partial derivatives using numerical techniques entails solving the system of ODEs several times. Some computational cost may be spared through taking advantage of the known relationship that *S* + *I* + *R* = 1.

**Figure 12.**
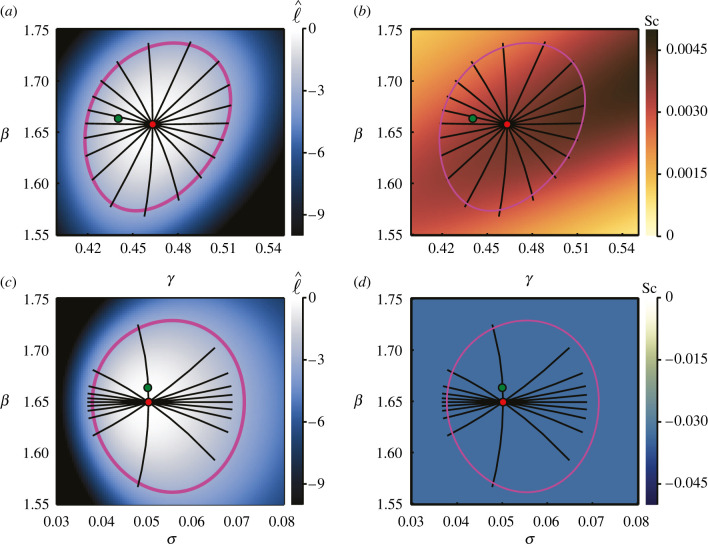
Inferring ***θ*** = (*β*, *γ*) in (*a*,*b*) and ***θ*** = (*β*, *σ*) in (*c*,*d*) for the SIR model with observations only on the number of infected individuals. Observations in the synthetic data occur at *T* = (4, 7, 10), with *N* = 10 observations per time point. True parameters, (*β*, *γ*, *σ*) = (1.66334, 0.44036, 0.05), are marked with green discs, with MLEs indicated using red discs. Magenta curves correspond to likelihood-based 95% confidence regions. Black lines are geodesic curves emanating from the MLEs, with lengths corresponding to a theoretical 95% confidence distance. Initial populations are as described in figure 11.

**Figure 13.**
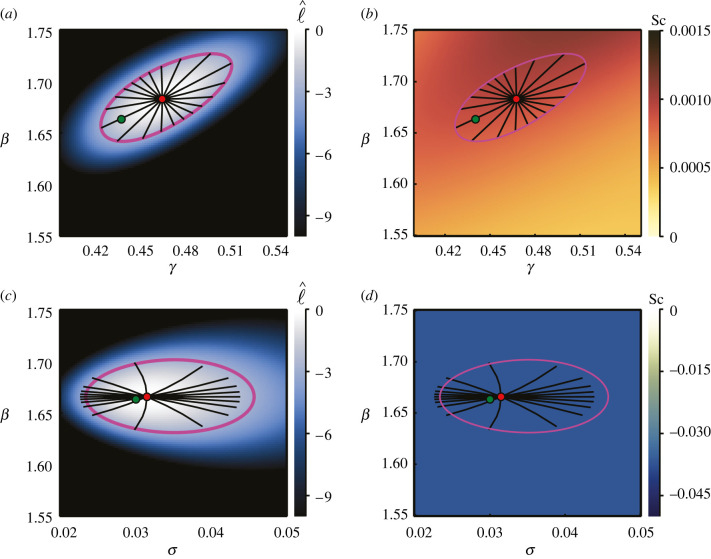
Inferring ***θ*** = (*β*, *γ*) in (*a*,*b*) and ***θ*** = (*β*, *σ*) in (*c*,*d*), with observations on all three variables, *S*, *I* and *R*. Observations in the synthetic data occur at *T* = (4, 7, 10), with three observations of each population at each time point; 27 observations in total, as depicted in figure 11*b*. True parameters, (*β*, *γ*, *σ*) = (1.66334, 0.44036, 0.03), are marked with green discs, with MLEs indicated using red discs. Magenta curves correspond to likelihood-based 95% confidence regions. Black lines are geodesic curves emanating from the MLEs, with lengths corresponding to a theoretical 95% confidence distance. Initial populations as described in figure 11.

For brevity, we restrict our investigation of the SIR model to the cases where ***θ*** = (*β*, *γ*) and ***θ*** = (*β*, *σ*). Results in figure 12 correspond to the case where observations pertain only to the number of infected individuals, while those in figure 13 are produced from data containing observations of all three populations. In both cases, the results for ***θ*** = (*β*, *σ*) align with those observed in previous results; the geodesics appear to define a marginally smaller area and are offset from the likelihood-based confidence regions in the direction of decreasing *σ* and the scalar curvature is the constant Sc = −1/*N*.

Regardless of whether we observe only the infected population or all populations, inferring ***θ*** = (*β*, *γ*) produces a non-constant positive scalar curvature. In figure 12*b*, where only *I* is observed, we see that the geodesics emanating from the MLE extend beyond the likelihood-based confidence region. This also occurs in figure 13*b*, where all three populations are observed, however it is difficult to perceive at this scale. Based on this result, and the observations involving negative scalar curvature when inferring *σ*, it might seem that positive scalar curvature produces geodesics that extend beyond corresponding likelihood-based confidence regions, whereas negative scalar curvature has the opposite effect. However, repeating the analysis with different synthetic datasets—generated from a different random seed—suggests that in some cases the geodesics will extend beyond the likelihood-based confidence regions, and in some cases they will fall short, however the scalar curvature remains positive in all cases.

### 3.4. Hypothesis testing

**Figure 14.**
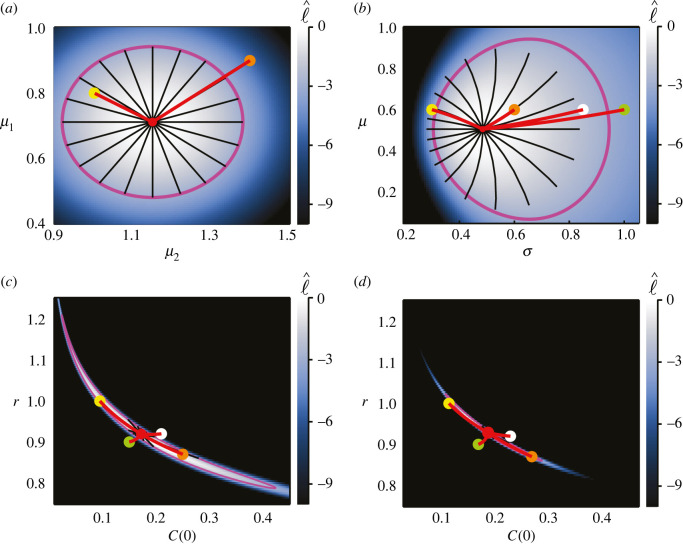
Example hypothesis tests for the: (*a*) univariate normal distribution, with ***θ*** = (*μ*, *σ*), $\;\hat{\theta }=(0.5050,0.4846)$; (*b*) multivariate normal distribution, with ***θ*** = (*μ*_1_, *μ*_2_), $\;\hat{\theta }=(0.7109,1.1498)$; logistic model with ***θ*** = (*r*, *C*(0)) in the high curvature region as considered in figure 9, with (*c*) *N* = (10, 10, 10), $\;\hat{\theta }=(0.9195,0.1723)$, and (*d*) *N* = (50, 50, 50), $\;\hat{\theta }=(0.9287,0.1682)$. In each case, we test several example hypotheses, ***θ***_0_, marked by coloured discs. Geodesics between the MLEs (red discs) and each ***θ***_0_ are shown in red. Magenta curves correspond to likelihood-based 95% confidence regions. Black lines are geodesic curves emanating from the MLEs, with lengths corresponding to a theoretical 95% confidence distance.

In figure 14, we present several example hypothesis tests, using both likelihood-ratio-based and geodesic-distance-based approaches, as outlined in §2. Test statistics and corresponding *p*-values for each hypothesis test are provided in table 1. For the multivariate normal distribution, where we observe that the endpoints of geodesics corresponding to a theoretical 95% confidence distance align closely with the likelihood-based 95% confidence regions, we find that the results of the hypothesis tests are near-identical. Further, the hypothesis test results are consistent with our interpretation of the 95% confidence regions; test points within the confidence regions have *p*-values greater than 0.05, while test points outside the confidence regions have *p*-values less than 0.05.

We also perform hypothesis tests for the logistic model in the high curvature region of parameter space. Like before, results are comparable for different numbers of observations at each time point, *N* = (10, 10, 10) and *N* = (50, 50, 50), as considered in figure 9. Even in this high curvature region, we find that the endpoints of geodesics corresponding to a theoretical 95% confidence distance very closely match the likelihood-based 95% confidence regions. This is again reflected in the results of the hypothesis tests, where very similar results are obtained from the likelihood-ratio-based hypothesis tests and the geodesic-distance-based hypothesis tests, even for relatively extreme ***θ***_0_. As the number of observations increases, we observe for each ***θ***_0_ considered that, in accordance with the confidence regions tightening, the test statistics increase and accordingly *p*-values decrease.

**Figure 15.**
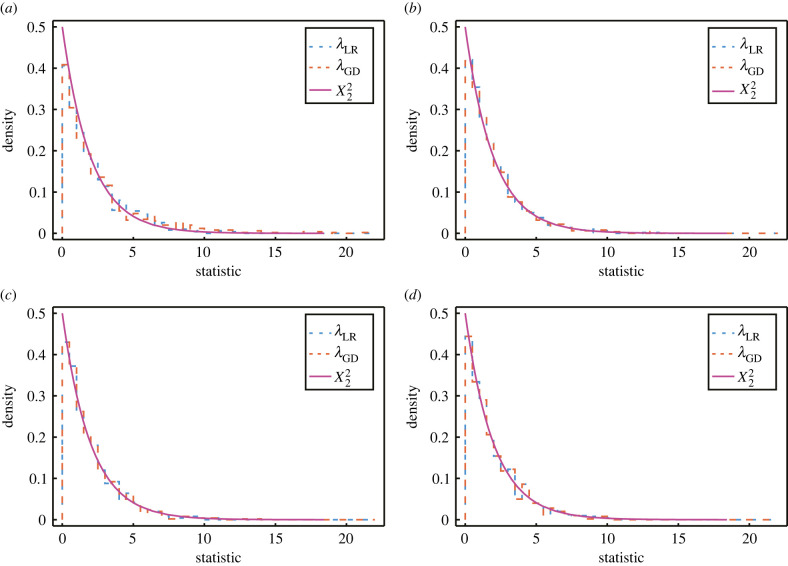
Step histograms show the density of the distribution of test statistics for each hypothesis testing approach, for (*a*,*b*): the univariate normal distribution with ***θ*** = (*μ*, *σ*), and (*c*,*d*): the multivariate normal distributions with ***θ*** = (*μ*_1_, *μ*_2_). Test statistics are computed from the true parameter values and the MLE, for 1000 sets of synthetic data. Datasets represented in (*a*,*c*) contain *N* = 10 observations, while in (*b*,*d*) *N* = 1000. Purple curves correspond to the density of the $\chi_{2}^{2}$ distribution, while blue dotted lines represent the likelihood-ratio-based test statistics and orange dashed lines represent the geodesic-distance-based test statistics.

As we are using synthetic data and know the true parameters, we can use hypothesis testing to pedagogically investigate Wilks’ theorem [36] and the asymptotic relationship given in (2.11). We generate 1000 synthetic datasets and for each dataset perform a hypothesis test for the true parameters. This is repeated for the univariate and multivariate normal distributions with *N* = 10 and *N* = 1000 observations. In figure 15, we present densities for both the likelihood-ratio-based and geodesic-distance-based test statistics, alongside the probability density of $\chi_{2}^{2}$. For the multivariate normal distribution with ***θ*** = (*μ*_1_, *μ*_2_), the density profiles for *λ*_LR_ and *λ*_GD_ are near-identical, as expected following the results in figure 14 and table 1. We also observe a good match between these profiles and $\chi_{2}^{2}$, even with just *N* = 10. For the univariate normal distribution with ***θ*** = (*μ*, *σ*), when *N* = 10 we observe differences between *λ*_LR_ and *λ*_GD_. Both profiles are similar to $\chi_{2}^{2}$, though there appears to be a higher density in the tails of the distributions of the test statistics. As the number of observations increases to *N* = 1000, the difference between *λ*_LR_ and *λ*_GD_ reduces significantly, and both closely match $\chi_{2}^{2}$.

**Table 1.** Hypothesis test results.

model	***θ***_0_	*λ*_LR_	*λ*_GD_	*p*_LR_	*p*_GD_
multivariate	(0.8,1.0)	3.3737	3.3737	0.1851	0.1851
normal	(0.9,1.4)	10.9297	10.9297	0.0042	0.0042
univariate	(0.6,0.3)	7.5051	5.1954	0.0235	0.0744
normal	(0.6,0.6)	1.0460	1.2201	0.5927	0.5433
	(0.6,0.85)	4.6134	6.5226	0.0996	0.0383
	(0.6,1.0)	6.9271	10.6665	0.0313	0.0048
logistic	(1.0,0.095)	2.7086	2.5798	0.2581	0.2753
(10, 10, 10)	(0.87,0.25)	1.6387	1.5626	0.4407	0.4578
	(0.92,0.21)	30.0130	29.9821	3.0391×10^−7^	3.0865×10^−7^
	(0.9,0.15)	56.2776	56.5328	6.0185×10^−13^	5.2969×10^−13^
logistic	(1.0,0.095)	31.3038	31.0222	1.5939×10^−7^	1.8349×10^−7^
(50, 50, 50)	(0.87,0.25)	4.2062	4.2276	0.1221	0.1208
	(0.92,0.21)	97.6247	97.5164	<10^−16^	<10^−16^
	(0.9,0.15)	368.1479	368.7335	<10^−16^	<10^−16^

From Wilks’ theorem [36] and (2.11), asymptotically 95% of the 95% confidence regions we construct should contain the true parameter values. We can determine what proportion of the likelihood-based and geodesic-distance-based 95% confidence regions that we construct contain the true parameter values using the information presented in figure 15. This is done by comparing the test statistics with the critical value, Δ_2,0.05_, from (2.3). For the multivariate normal distribution with *N* = 10 we find that 95.7% of the likelihood-based and geodesic-distance-based confidence regions contain the true parameter values. With *N* = 1000 we find that 94.8% contain the true parameters, approaching the theoretical 95%. For the univariate normal distribution with *N* = 10 we find that 93.2% of the likelihood-based confidence regions contain the true parameter, while only 88.0% of the geodesic-distance-based confidence regions contain the true parameters. With *N* = 1000, we find that 95.2% of the likelihood-based confidence regions and 95.1% of the geodesic confidence regions contain the true parameters.

## 4. Discussion

Parameter estimation is wrought with challenges relating to the availability and quality of experimental or field data [8,9,11,12]. This prompts a strong consideration of uncertainty quantification to support point estimation of model parameters [13]. In this section, we discuss the results presented in §3. We highlight opportunities for application of information geometry techniques, including geodesic curves and scalar curvature, to supplement traditional maximum-likelihood-based parameter inference and uncertainty quantification. We conclude by outlining areas for further investigation.

Even for relatively small sample sizes, we observe good correspondence between the likelihood-based 95% confidence regions and the endpoints of geodesic curves corresponding to a theoretical 95% confidence distance, in accordance with the asymptotic relationship described in equation (2.11), particularly when estimating model parameters. When estimating standard deviation, as outlined in §3, geodesics appear to suggest a tighter confidence region and appear to be biased towards parameter space with smaller standard deviation. We observe this effect decreasing as the number of observations increases, in line with the known underestimation bias of minimum-likelihood estimates of variance [36]. The misalignment of likelihood-based confidence regions and geodesic endpoints appears to occur more frequently in examples with non-zero scalar curvature, although we observe a good match in figure 9 despite the non-constant scalar curvature.

Visualizing the scalar curvature throughout a parameter space can indicate areas where there may be issues with identifiability. Areas with significant non-constant scalar curvature can suggest a complicated relationship between parameters in terms of the normalized log-likelihood, such as the hyperbolic confidence region observed in figure 9. However, it is possible to produce examples, such as figure 8*c*,*f*, where there is practical non-identifiability despite zero scalar curvature everywhere. Although we do not show it here, for the logistic model with ***θ*** = (*r*, *K*) in the region of parameter space where *C*(0) ≈ *K*, computation of the scalar curvature breaks down as the Fisher information matrix becomes singular. Here, it may be obvious that we cannot identify the growth rate, *r*, from a process that is initialized at its steady state (*C*(0) = *K*). However, observing this behaviour in general may help to detect issues with identifiability, particularly for models without analytical solutions.

The information geometry techniques we discuss are primarily implemented numerically; as such there is a computational cost to consider. For the normal distributions and population growth models in this work, where analytical solutions are available, the information geometry techniques are not disproportionately more computationally expensive than the traditional likelihood-based inference and confidence regions. Examples such as the SIR model, where no analytical solution is available, represent a significantly greater computational burden. However, this impacts both the likelihood-based inference and information geometry techniques as the underlying system of ODEs, for example equation (3.5), must be solved numerous times. The computational cost associated with the information geometry techniques depends significantly on the desired resolution for the scalar curvature surface, and on the number of geodesic curves. A suitable approach may be to first compute the scalar curvature on a coarse grid to identify areas of interest to investigate with a refined grid. Further, the geodesic curves and scalar curvature computations are highly amenable to parallelization, which can significantly reduce computation time. This computational cost will generally pale in comparison with the costs associated with collecting experimental or field data, and may be easily justified if the information geometry techniques are used to guide data collection. If information geometric analysis identifies a region of parameter space with significant non-constant scalar curvature for a model, such as in figure 9, and practitioners have a prior expectation that the true parameter values fall somewhere within this region, this may indicate that a greater quantity or quality of data is needed to improve identifiability for that particular model. Alternatively, such analysis may guide practitioners in choosing favourable experimental conditions; for example in cell culture experiments, where it is possible to vary the initial cell seeding density [1]. Experimental design is a process wherein experiments are performed or simulated iteratively with perturbations, such that some measure of information is maximized. Through this process, the most informative experiments are identified, facilitating design of optimal experimental protocols [96–98]. Common to these approaches is the importance of quantifying and comparing information. While we do not consider optimal experimental design in this work, there is potential to incorporate information geometric techniques in the experimental design process as a means of comparing information between experimental perturbations. This is an area for further investigation. Although we focus on how information geometry can supplement traditional maximum-likelihood-based inference and uncertainty quantification, primarily through visualization, it should be noted that concepts from information geometry have also found application in the inference context from a computational efficiency standpoint. For example in Bayesian inference, by defining Monte Carlo sampling methods on a Riemann manifold, the geometric structure of the parameter space can be exploited [99]. Simulated paths across the manifold automatically adapt to local structure, facilitating efficient convergence, even in higher dimensions and in the presence of strong correlation [99,100]. Concepts from information geometry, including geodesic curves, are also implemented in methods for model reduction [101]. These applications of information geometry techniques to improve computational algorithms highlight further utility of geometric concepts for inference in higher dimensions, beyond that which we demonstrate through visualization in this work. Geodesics can be used to measure the distance between probability distributions. As demonstrated in §3, it is possible to perform hypothesis tests based on geodesic distance [48,51,78]. The approach for performing a hypothesis test is to solve a boundary value problem to find the geodesic connecting two points in parameter space, and use the corresponding geodesic distance to compute a test statistic. For the examples considered in this work, such boundary value problems are readily solved numerically using standard techniques, such as those included in the Julia package DifferentialEquations.jl [83]. Careful numerical handling may be required for geodesic curves close to boundaries of parameter space. For more complicated examples, particularly those in high-dimensional manifolds, achieving converging solutions to geodesic boundary value problems can prove challenging. There is scope for a review of the different numerical methods for solving boundary value problems, with a particular focus on their applicability to solving geodesic boundary value problems for hypothesis testing in high-dimensional manifolds.

In this work, we only consider models that admit unimodal likelihoods. In cases where the likelihood is multimodal, provided that we are able to obtain the Fisher information required to compute the Christoffel symbols, we are still able to compute the scalar curvature and perform hypothesis tests based on geodesic distance. With multimodal likelihoods, it would not be possible to construct confidence regions from geodesics emanating from the MLE. Although, we note that constructing confidence regions for multimodal likelihoods is also problematic with traditional likelihood-based inference methods. There are several avenues for future research in this area. Here, we consider two-dimensional manifolds to facilitate convenient visualization; however, the inference and information geometry techniques are general, and can be readily applied to higher dimensional manifolds [36,59], albeit with increased computational cost. Extending this analysis to three dimensions would enable consideration of situations where there is scalar curvature associated both with the variability of the observation process, *σ*, and also with interactions between model parameters; for example, it may be insightful to consider ***θ*** = (*β*, *γ*, *σ*) for the SIR model, where we associate a constant negative scalar curvature with *σ* and non-constant positive scalar curvature due to interactions between *β* and *γ*. In three dimensions, likelihood-based confidence regions can be visualized as a series of two-dimensional slices oriented in three-dimensional space [1]; this technique could be applied to visualize slices of the scalar curvature in three dimensions. One approach for visualization in higher dimensions is to produce an ensemble of these two- or three-dimensional confidence regions for various combinations of parameters of interest, with other parameters fixed at their MLEs. Alternatively, in higher dimensions it may be more appropriate to use non-visual techniques, such as hypothesis testing.

While we have considered ODE models, there is appetite in the literature for parameter estimation, uncertainty quantification and identifiability analysis for more complicated models, including partial differential equations, stochastic differential equations (SDEs) and delay differential equations [19,102,103]. This appetite extends to non-differential-equation-based models, including agent-based models [104] and network models [105]. A natural extension of this work is to present examples demonstrating how the information geometry techniques can be applied to these more complicated models. This will introduce new challenges, though it may be possible to leverage existing techniques; for example, linear noise approximation may be used to obtain a representation of the Fisher information matrix for SDEs [24]. Further, we fix *σ* across observation times, model parameters and populations. However, the techniques presented in this work can be generalized to handle data with non-constant variance [72]; the expression for the Fisher information matrix given in equation (1.4) can be extended to account for a parameter-dependent covariance matrix [106]. Investigation of examples paralleling those in §3, but with non-constant standard deviation, may prove insightful.

Here, the Fisher information defines a Riemann metric on the statistical manifold. For some inference problems, it is not practical to obtain the Fisher information. Where the Fisher information is not available, the sample-based *observed information*—computed as negative the Hessian of the log-likelihood function, or via Monte Carlo methods—may be available [107,108]. The observed information has been demonstrated to equip a manifold with an *observed geometric structure* akin to the *expected geometric structure* associated with the Fisher information [109]. Further work could identify the viability of the techniques presented here in situations where only the observed information is available, particularly for local approximation about the MLE.

## Data Availability

Data and code are made available on GitHub.
